# Effects of Selenium Supplementation on Selenoprotein Gene Expression and Response to Influenza Vaccine Challenge: A Randomised Controlled Trial

**DOI:** 10.1371/journal.pone.0014771

**Published:** 2011-03-21

**Authors:** Andrew J. Goldson, Susan J. Fairweather-Tait, Charlotte N. Armah, Yongping Bao, Martin R. Broadley, Jack R. Dainty, Caroline Furniss, David J. Hart, Birgit Teucher, Rachel Hurst

**Affiliations:** 1 Institute of Food Research, Norwich Research Park, Norwich, United Kingdom; 2 Norwich Medical School, University of East Anglia, Norwich, United Kingdom; 3 School of Biosciences, University of Nottingham, Sutton Bonington, Loughborough, United Kingdom; University of California Los Angeles, United States of America

## Abstract

**Background:**

The uncertainty surrounding dietary requirements for selenium (Se) is partly due to limitations in biomarkers of Se status that are related to health outcomes. In this study we determined the effect of different doses and forms of Se on gene expression of selenoprotein S (SEPS1), selenoprotein W (SEPW1) and selenoprotein R (SEPR), and responses to an immune function challenge, influenza vaccine, were measured in order to identify functional markers of Se status.

**Methods and Findings:**

A 12 week human dietary intervention study was undertaken in 119 volunteers who received placebo, 50, 100 or 200 µg/day Se-enriched yeast (Se-yeast) or meals containing unenriched or Se-enriched onions (50 µg/day). Gene expression was quantified in RNA samples extracted from human peripheral blood mononuclear cells (PBMC's) using quantitative RT-PCR. There was a significant increase in SEPW1 mRNA in the Se-enriched onion group (50 µg/day) compared with the unenriched onion group. SEPR and SEPW1 did not change significantly over the duration of the supplementation period in the control or Se-yeast groups, except at week 10 when SEPW1 mRNA levels were significantly lower in the 200 µg/day Se-yeast group compared to the placebo group. Levels of SEPS1 mRNA increased significantly 7 days after the influenza vaccine challenge, the magnitude of the increase in SEPS1 gene expression was dose-dependent, with a significantly greater response with higher Se supplementation.

**Conclusions:**

This novel finding provides preliminary evidence for a role of SEPS1 in the immune response, and further supports the relationship between Se status and immune function.

**Trial Registration:**

ClinicalTrials.gov [NCT00279812]

## Introduction

Selenium (Se) is involved in a wide variety of functions in the human body [1] and has been reported to reduce the incidence and mortality risk of prostate, colon and lung cancer [2]-[6]. Se also has an important role in the function of the immune system [7] as it has been demonstrated to be improved in Se-deficient populations given Se supplements [7], [8]. In several European populations Se intakes are below recommended intakes [9] and therefore there is a need to evaluate the consequences of sub-optimal status to enable public health policies to be developed [10].

Long-term status may be assessed from erythrocyte, hair or toenail Se content. However, such measures have no universally accepted reference ranges due to large geographical variations in Se intake [10]. Plasma Se is commonly used as a short-term measure of status but different forms of dietary Se result in different responses in plasma Se concentration [11] and the Se present in the circulation may not be available for incorporation into functional proteins [12]; organic forms such as selenomethionine may be readily incorporated into plasma albumin or erythrocyte haemoglobin whereas inorganic forms may not [13]. Measurement of the expression of individual selenoproteins may therefore provide a more appropriate measure of Se status [14]. The human selenoproteome is comprised of 25 selenoproteins [15] and it is therefore likely that the combination of a number of key selenoproteins will determine Se status [10], [16].

At present, recommendations for Se intake are based on maximising plasma glutathione peroxidase (GPx3) levels [17] but there is considerable debate as to the appropriateness of this endpoint [10]. Red blood cell glutathione peroxidase (GPx1) has proved useful for identifying individuals/populations with low Se status, but as with plasma GPx3, the enzyme activity plateau is reached relatively quickly as Se intake is increased [12]. Glutathione peroxidase 4 (GPx4) has also been proposed as a possible functional marker of Se status [18], but there is significant heterogeneity in the data from published studies to date [19] and the activity reaches a plateau at a relatively low Se intake, similar to GPx1. Selenoprotein P is the main Se-containing protein in human plasma, and is a reliable biomarker for Se-deficient populations, with a higher plateau level than some of the glutathione peroxidases [20]. Other less well studied selenoproteins, such as selenoprotein W (SePW1), selenoprotein S (SePS1) and selenoprotein R (SePR), are potential candidates as novel biomarkers. SePW1 and SePR are reported to exhibit antioxidant activity [21], [22]; *in vitro* over-expression of SePW1 in H1299 cells resulted in reduced susceptibility to oxidative challenge by hydrogen peroxide [21], and SePR catalyses thioredoxin-dependent methionine-*R-* sulfoxide reduction [22]. SePS1 has been identified as a protein associated with the endoplasmic reticulum which maintains lumen homeostasis by removal of misfolded proteins to the cytosol for polyubiquitination and proteasomal degradation [23].

The aims of this study were to measure the expression of SEPS1, SEPR and SEPW1 after supplementation with different forms and doses of Se, and the changes in response to influenza vaccine (as an immune function challenge). The expression levels were quantified and compared with ‘classical’ biomarkers of Se status. This is the first report of novel analysis of key Se-responsive genes in response to supplements of Se-enriched yeast (Se-yeast) and Se-enriched onions, and the effect of an immune function challenge using influenza vaccine.

## Methods

The protocol for this trial and supporting CONSORT checklist are available as supporting information; see Checklist S1 and Protocol S1.

### Subjects and study design

A dietary intervention was undertaken, using a parallel design, in adults with suboptimal Se status, defined by low plasma Se concentration (<110 ng/ml). This study was part of a randomised, double-blind, placebo-controlled trial from which the results for the use of plasma selenoprotein P as a biomarker have previously been published [24]. Recruitment ran from May until the following February in 2005, 2006 and 2007. This was related to the timing of the influenza vaccine administration. For ethical and vaccine availability reasons the vaccine had to be administered only during September to April; volunteers were therefore recruited in May and began the study from July onwards to coincide with the vaccination period. Recruitment stopped at the beginning of February each year so that volunteers completed the study before the end of April. A pre-study health screen was undertaken to assess basic blood chemistry and Se status in each potential volunteer; the full list of exclusion criteria are given in Hurst *et al* [24]. A total of 119 free-living, non-smoking men and women, aged 50–64 y, completed the study (Figure S1). Each subject was randomly assigned to one of six groups and given tablets containing either 50 (n = 18), 100 (n = 21) or 200 (n = 23) µg Se/day Se-yeast, meals made with Se-enriched onions containing 50 µg Se/day (n = 18) or unenriched onions (n = 17), or placebo tablets (n = 20) for a period of 12 weeks. For the allocation of volunteers, a computerised random number generator was used (URL: http://www.randomizer.org/form.htm). The tablets were provided using a double blind design, as were the 2 onion groups. The double blind coding was not revealed until the completion of the final data analysis. Volunteer compliance to the interventions (for both the tablets and onion meal groups) was monitored by self-administered tick sheets and in addition, for the supplement groups by counting the number of tablets returned at the 6 and 12 week time points. At week 10 the participants were vaccinated intra-muscularly with a trivalent influenza vaccine, developed from World Health Organisation guidelines. Blood samples (65 ml) were drawn from an antecubital vein in each volunteer's forearm at week 0, 6 and 10 for pre-vaccination samples and at week 11 and 12 for post-vaccination samples.

### Platelet isolation and preparation of enzyme extracts

Platelets were isolated by centrifugation [18] from 8 ml whole blood collected in citrate coated polypropylene tubes (Sarstedt, Germany, http://www.sarstedt.com) and were subsequently frozen in 0.32 M sucrose solution, with controlled temperature gradient freezing to −80°C. When required for batch analysis of GPx1 and GPx4 activity, enzyme extracts were prepared using ice-cold protein extraction cell lysis buffer (100 mM Tris-HCl solution pH 7.4, 1 mM dithiothreitol DTT, 0.1% Triton X100 and protease inhibitor) and probe sonication with a sonicator (Status 70, MS 72, Bandelin, Germany, http://www.bandelin.com). Samples were maintained below 4°C during sonication, and then centrifuged at 12000g at 4°C for 10 min, the supernatants were stored on ice and GPx1 activity was quantified within 4 hours. Total protein concentrations were determined using the method of Bradford [25] with HSA as a standard.

### Red blood cell isolation and preparation of enzyme extracts

Approximately 10 ml of whole blood was collected in a BD Vacutainer™ EDTA tube (BD Medical, Cowley, UK, http://www.bd.com/). After centrifugation for 10 min at 1500 × g, 20°C the plasma layer and buffy coat were removed and the remaining erythrocytes washed twice with ice-cold PBS. Erythrocytes were diluted with one volume of ice-cold PBS and stored at −80°C. Enzyme extracts were prepared in batches from frozen erythrocyte samples, were stored on ice and used within 4 hours for the determination of Se dependent GPx1 activity. Haemoglobin (Hb) was quantified in the erythrocyte enzyme extracts using the method described by Drabkin [26].

### Se-dependent GPx1 activity in red blood cells and total GPx1 activity in platelets

Glutathione peroxidase 1 activities in erythrocyte and platelet samples were quantified using a spectrophotometric method [27]. The assay reaction mixture contained 100 mM Tris-HCl pH 7.4, 3 mM glutathione, 0.25 mM NADPH, 1U glutathione reductase and triton X100 (0.1%). A high-throughput 96-well enzyme assay [28] was used to analyse samples and controls in triplicate, with tert-butyl-hydroperoxide or cumene hydroperoxide as the substrates for Se-dependent GPx1 activity or total GPx1 activity respectively [29]. The rate of decrease in absorbance at 340 nm was monitored at 37°C for 15 min with measurements taken every 10 seconds. The GPx1 activity was calculated from the initial rates of reaction as described by Paglia and Valentine [27]. One unit (U) of glutathione peroxidase activity is defined as 1 µmol of NADPH oxidised per minute.

### GPx4 activity in platelets

Preparation of the reaction substrate 1-palmitoyl-2-(13-hydroperoxy-cis-9, trans-11-octadecadienoyl)-l-3-phosphatidylcholine (PLPC-OOH) was as described by Bao *et al* [30]. The assay reaction mixture included 0.1 M Tris-HCl (pH 7.4), 2 mM EDTA, 1 mM sodium azide (NaN_3_), 0.12% Triton X-100, 3 mM glutathione and an appropriate amount of platelet sample in 500 µl. The mixture was incubated at 37°C for about 3 min and the reaction was started by the addition PLPC-OOH to produce a final concentration of 25 µM. The reaction was stopped by adding ice cold acetonitrile and then centrifuged at 12,000g at 4°C for 3 min to prepare for HPLC analysis. Separation of the product (PLPC-OH) from the substrate (PLPC-OOH) was carried out using a Gemini 5 m C18 110A column (250×4.6 mm) (Phenomenex, Macclesfield, UK, http://www.phenomenex.com) at 30°C. The mobile phase was a mixture of acetonitrile-methanol-water (50:49.5:0.5, v/v/v) containing 10 mM choline chloride. The flow rate was 0.5 ml/min and the UV detector wavelength was set at 232 nm. GPx 4 activity was calculated from the PLPC-OOH and PLPC-OH peaks as described [30] and expressed per mg total protein.

### Plasma Se

Approximately 10 ml whole blood was collected in sodium heparin trace element free tubes (BD Medical, Cowley, UK). After centrifugation for 10 minutes at 1500g, 20°C the plasma was removed and stored at −80°C in trace element free tubes (BD Medical, Cowley, UK, http://www.bd.com/). All samples were analysed in duplicate in batches and a reference serum sample (Seronorm, Norway, http://www.sero.no/) was analysed and used as a quality control check on each batch. Rhodium was added as an internal standard and Se concentrations were determined using a 7500ce inductively coupled plasma mass spectrometer (Agilent Technologies, Santa Clara, USA, http://www.agilent.com/) fitted with a dynamic reaction cell operating in the hydrogen mode. Se was determined by monitoring at m/z 76, 77 and 78 for Se and m/z 103 for the rhodium internal standard against Se standards of 0, 0.5, 1.0, 2.0, 5.0 and 10.0 ng/ml.

### Peripheral blood mononuclear cell (PBMC) isolation and preparation of total RNA for quantitative real time RT-PCR

Approximately 8 ml of whole blood was collected in a BD Vacutainer™ CPT™ tube (BD Medical, Cowley, UK, http://www.bd.com/). Blood samples were processed within 30 min and PBMC's isolated according to the manufacturer's instructions [31]. Isolated PBMC's were lysed and homogenised using a Qiashredder column and total RNA was then isolated using RNeasy mini kit according to manufacturer's instructions (Qiagen, Crawley, UK, http://www.qiagen.com). RNA was eluted from the binding column using 50 µl of RNase-free water. Ribonuclease inhibitor (Promega, Madison, USA, http://www.promega.com) was added immediately (20 U/preparation) and samples were stored at −80°C. Total RNA yield was determined using a NanoDrop ND-1000 spectrophotometer (Thermo Fischer Scientific, Breda, Netherlands, http://www.thermofisher.com) and purity assessed by the ratio of absorbance at 260 and 280 nm.

### Gene expression (SEPS1, SEPR, SEPW1) using quantitative real time RT-PCR

Determination of mRNA levels was performed by quantitative real-time reverse transcription-PCR (RT-PCR) using ABI Prism 7300 Sequence Detection System (Applied Biosystems, Warrington,UK, http://www.appliedbiosystems.com). Primers and fluorogenic probes (5′ FAM- 3′ TAMRA) were designed across exon-exon boundaries using Primer Express Software (Operon, Cologne, Germany, http://www.operon.com/) (Table 1). Oligomer specificity was checked using NCBI BLAST (http://blast.ncbi.nlm.nih.gov/Blast.cgi) searches to confirm no sequence homologies with unrelated targets. Amplification products for each primer and probe set were run on 10% TBE Novex gels (Invitrogen, Paisley, UK, http://www.invitrogen.com) to verify the sizes of the resulting amplicons. RT-PCR reactions were performed in 96 well plates using TaqMan one-step RT-PCR master mix reagent kit (Applied Biosystems, Warrington,UK, http://www.appliedbiosystems.com) in a total volume of 25 µl/well consisting of 100–200 nM probe, 200–400 nM forward and reverse primers and 10 ng RNA. TaqMan RT-PCR conditions were as follows: 48°C for 30 min, 95°C for 10 min then 40 cycles of 95°C for 15 s and 60°C for 1 min. Gene expression was quantified using the relative standard curve method [32]. A master stock of total RNA was extracted from the pooled blood of 3 individuals. Standard curves using the master RNA stock were included on every plate, each time using triplicate replicates for each amount. The range of the standard curve encompassed the observed range of sample values. *β*-glucuronidase (GUSB) was measured as a reference gene [33], [34] (Table 1).

**Table 1:** Primer and probe sequences used for real-time reverse transcription PCR reactions.

GeneAccesssion No.	Sequence (5′-3′)
**SEPW1**	Forward primer AGGCCACCGGGTTCTTTG
NM_003009	Reverse primer CGTAGCCATCGCCTTTCTTC
	Probe *FAM*-AGAGTGAATCAACTTCCCGGCTACCATCA-*TAMRA*
**SEPS1**	Forward primer AGCCCCAGGAGGAAGACAGT
NM_018445	Reverse primer TCCCCGCAAAGGCTTTCT
	Probe *FAM*-ACTTCATCTGTCCTGAAACGGAAATCGGA-*TAMRA*
**SEPR**	Forward primer GCGTCCGGAGCACAATAGAT
NM_016332	Reverse primer TGGCCCAACCCATTGC
	Probe *FAM-*CACAGGACACCTTCAAGGCTTCA-*TAMRA*
**GUS**	Forward primer TTGGCAGTGCCCATTCCT
NM_000181	Reverse primer GTAGCCCCCCTCATGCTCTAG
	Probe *FAM*-CCCATTCACCCACACGATGGCA-*TAMRA*

### Ethics

Governance and ethical approvals for this study were obtained from the Institute of Food Research Governance Committee, the East Norfolk and Waveney Research Governance Committee and the Norwich Local Ethics Committee (Text S1). Written informed consent was obtained from each participant.****

### Statistics

The primary endpoints of the study were changes in gene expression of SEPS1, SEPW1 and SEPR in response to dietary Se supplementation comparing baseline values with those at weeks 6, 10, 11 and 12. The secondary endpoints were changes in SEPS1, SEPW1 and SEPR between vaccine administration at week 10 to weeks one and two post-vaccination (weeks 11 and 12). Statistical analyses were performed using the R data analysis software [35]. Standard ANOVA models and mixed-effects models were employed to analyse these data. The main effects tested in the models were supplementation group and time; interactions of these terms were also tested. For all models, diagnostics were checked to determine if data transformations, outlier omissions, or alternative non-parametric models were required. All results from the models were considered significant if P<0.05. When a factor in an ANOVA was significant, a Tukey's honest significant difference post-hoc test was applied. When a factor in the mixed-effects models showed a significant effect, contrasts between levels in the factor were used to estimate whether the pairwise differences were significant. Adjustments for multiple testing were made for all post-hoc tests.

## Results

### Blood analysis

Red cell count, white cell count, Hb, haematocrit, mean cell Hb and platelet count did not significantly change over the duration of the intervention. The mean red blood cell count was 4.6±0.48×10^12^/ L and ranged from 1.06 to 6.07×10^12^/ L, White blood cell counts were 5.3±1.3×10^9^/ L and ranged from 2.5–10.6), Hb (mean: 14.0±1.1 g/dL, range: 11.7–19.7), haematocrit (mean: 41±3% range: 34–51%), mean cell Hb (mean:30.6±2.5 pg range:12.1–58.7) and platelet count (mean:254±56×10^9^/ L range: 138–463).

### Effect of Se supplementation on Se-dependent GPx1 activity in red blood cells and total GPx1 activity in platelets

Se-dependent GPx1 activity was quantified in erythrocyte samples at week 0 and 12, the mean activities are displayed in Table 2. A significant effect of time was identified by ANOVA analysis for the erythrocyte GPx1 activity in the Se-yeast groups. Post-hoc analysis revealed a significantly greater erythrocyte GPx1 activity at week 12 compared to baseline week 0 (P<0.001). However, there were no significant effects of Se-yeast dose or Se-enriched onions on erythrocyte GPx1 activity compared to the placebo and unenriched onion groups respectively. There were also no significant differences in total GPx1 activity in platelets in the Se supplemented groups compared with the control groups. Over the duration of the intervention, time was found to have a significant effect on platelet total GPx1 activity. Post-hoc analysis showed this to be due to an increase in activity in only the 100 µg/day Se-yeast group at week 12 compared with baseline, week 0 (P<0.001). There were no significant effects of the influenza vaccine on platelet total GPx1 activity, week 10 compared with week 12 data, for any of the groups.

**Table 2:** Se-dependent glutathione peroxidase 1 activity in erythrocytes, total glutathione peroxidase 1 and Se-dependent glutathione peroxidase 4 activities in platelets: mean values at 0, 6, 10 and 12 weeks of supplementation and comparison of Se-yeast and Se-enriched onion meals with the placebo and unenriched onion groups respectively^1^.

	Se-dependent glutathione peroxidase 1 activity in erythrocytes (µmol/min per g Hb)		Total glutathione peroxidase 1 activity in platelets (µmol/ min per mg protein)				Se-dependent glutathione peroxidase 4 activity in platelets (µmol/min per mg protein)^2^			
Time (weeks)	0	12	0	6	10	12	0	6	10	12
Placebo (n = 20)	44.2±12.1 (34.4–82.9)	47.8±11.3 (32.8–75.7)	0.28±0.08 (0.13–.39)	0.29±0.11 (0.15–0.53)	0.26±0.09 (0.11–0.41)	0.29±0.07 (0.09–0.40)	8.6±5.0 (3.0–19.1)	11.1±8.4 (2.5–31.9)	9.1±4.6 (4.1–18.0)	9.4±6.4 (2.9–15.1)
50 µg/day Se-yeast (n = 18)	49.6±10.3 (34.4–65.2)	53.0±13.7 (35.0–78.9)	0.29±0.13 (0.16–0.44)	0.32±0.16 (0.15–0.64)	0.29±0.13 (0.15–0.49)	0.34±0.16 (0.12–0.72)	9.1±6.5 (2.5–24.1)	11.3±7.0 (3.3–29.2)	12.3±6.5 (5.3–23.1)	10.6±6.5 (2.5–24.0)
100 µg/day Se-yeast (n = 21)	46.3±11.7 (26.6–74.1)	48.4±14.1 (24.5–82.2)	0.25±0.13 (0.10–0.52)	0.28±0.12 (0.14–0.59)	0.27±0.14 (0.14–0.57)	0.34±0.15** (0.23–0.68)	8.4±3.9 (2.7–15.6)	12.0±9.4 (2.6–36.8)	10.9±5.8 (3.4–18.7)	11.8±7.1* (5.0–26.6)
200 µg/day Se-yeast (n = 23)	47.9±15.2 (27.1–76.1)	49.8±16.2 (28.4–83.2)	0.26±0.08 (0.11–0.40)	0.29±0.11 (0.16–0.58)	0.29±0.10 (0.17–0.50)	0.29±0.09 (0.17–0.51)	10.8±6.8 (2.5–19.1)	11.5±6.2 (0.4–22.0)	12.4±7.6 (3.4–30.7)	12.8±7.6 (2.5–28.9)
Unenriched onions (n = 17)	42.6±10.9 (19.7–63.7)	43.0±11.5 (19.9–64.7)	0.31±0.10 (0.12–0.50)	0.31±0.11 (0.13–0.49)	0.31±0.09 (0.15–0.48)	0.35±0.15 (0.18–0.67)	10.5±5.7 (3.6–23.2)	13.0±9.6 (6.0–44.5)	11.8±4.0 (6.6–20.2)	11.0±5.0 (1.4–19.1)
Se-enriched onions 50 µg/day (n = 18)	49.4±16.5 (24.0–87.3)	55.6±21.6 (24.3–106.4)	0.33±0.12 (0.18–0.54)	0.35±0.14 (0.16–0.59)	0.33±0.12 (0.09–0.50)	0.35±0.13 (0.15–0.67)	5.4±3.9 (0.4–13.1)	7.6±7.7 (0.8–26.4)	6.5±5.1^#^ (0.7–17.0)	7.0±6.7 (1.6–23.7)

^*1*^All values are means ± SDs; ranges in parentheses. Se-dependent activities in erythrocyte samples were determined using tert-butyl hydroperoxide as the substrate in the enzyme assay. Total GPx activities in platelet samples were determined using cumene hydroperoxide as the substrate in the enzyme assay [27], [29].

^*2*^numbers (n) for the GPx4 data are as detailed in the text; placebo (n = 11), 50, 100 and 200 µg/day (n = 12, 14 and 19 respectively), unenriched (n = 14) and Se-enriched (n = 9) due to undetectable GPx4 activity in the platelet enzyme extract compared with the control enzyme extraction buffer. * P<0.05 for the comparison of Time = 12 to Time = 0; ** P<0.005 for the comparison of Time = 12 to Time = 0; # P<0.005 for the comparison with unenriched onion.

### Effect of Se supplementation on GPx4 activity in platelets

The effects of the intervention on GPx4 activity are shown in Table 2. No significant differences were observed when comparing the Se-yeast groups with placebo group with respect to GPx4 activity. Overall, the Se-enriched onion group had lower GPx4 activity compared with the control unenriched onion group, which was largely attributed to the significantly lower (P<0.005) GPx4 activity at week 10 in the Se-enriched onion group than that of the unenriched onion group. It should be noted that some data are missing for samples where the activity of the enzyme extract in the platelet sample was less than the control enzyme extraction buffer which resulted in fewer data points in each of the groups (placebo n = 11; 50 µg/day Se-yeast n = 12; 100 µg/day Se-yeast n = 14; 200 µg/day Se-yeast n = 19; control unenriched onion n = 14; 50 µg/day Se-enriched onion n = 9). A significant effect of time on GPx4 activity was identified using ANOVA and post-hoc analysis showed a statistically significant (P<0.05) increase in platelet GPx4 activity at week 12 compared with week 0 only for the group that received 100 µg/day Se-yeast (Table 2). There were no significant effects of the influenza vaccine on platelet GPx4 activity, week 10 compared with week 12 data, for any of the groups.

### Plasma Se

**Figure 1:**
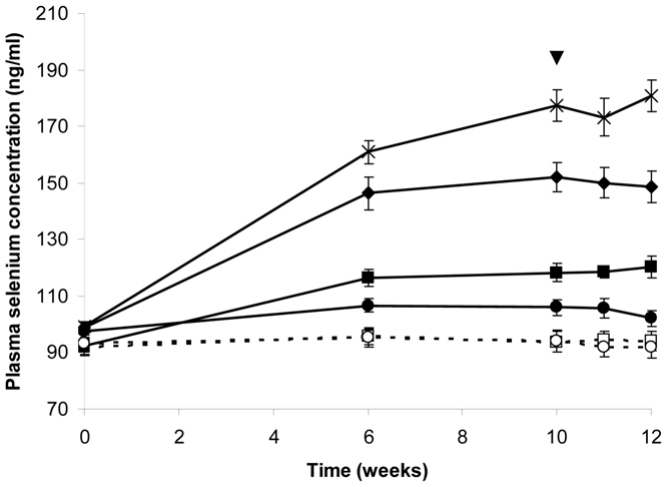
Plasma Se concentration over the 12 week intervention period for all groups. Values are means ± SEM. □ Placebo (n = 20); ▪ 50 µg/day Se-yeast (n = 17); ♦ 100 µg/day Se-yeast (n = 21); ×200 µg/day Se-yeast (n = 23); ○ Unenriched onions (n = 16); • 50 µg/day Se-enriched onion meals (n = 18). ▴ denotes influenza vaccine challenge administered at week 10 of the study. Plasma Se concentration data displayed for weeks 11 and 12, one and two weeks post-vaccination, are novel data. To display the effect of Se supplementation over the duration of the intervention period and to illustrate the plasma Se concentration before and after vaccination, data from time points baseline, week 6 and 10 are reproduced with permission of Hurst *et al*. 2010 [24].

Plasma Se concentration increased significantly in the Se-yeast groups compared to the placebo group up to week 10 as reported previously [24]. Previously unreported data (plasma Se concentration 1 and 2 weeks following influenza vaccination) show no significant change in any of the groups (Figure 1) which indicates that the volunteers had reached steady state Se status by week 10 and that the administration of influenza vaccine had no significant effect on plasma Se concentration.

### SEPW1, SEPR and SEPS1 gene expression in response to Se supplementation and effect of influenza vaccine challenge

**Figure 2:**
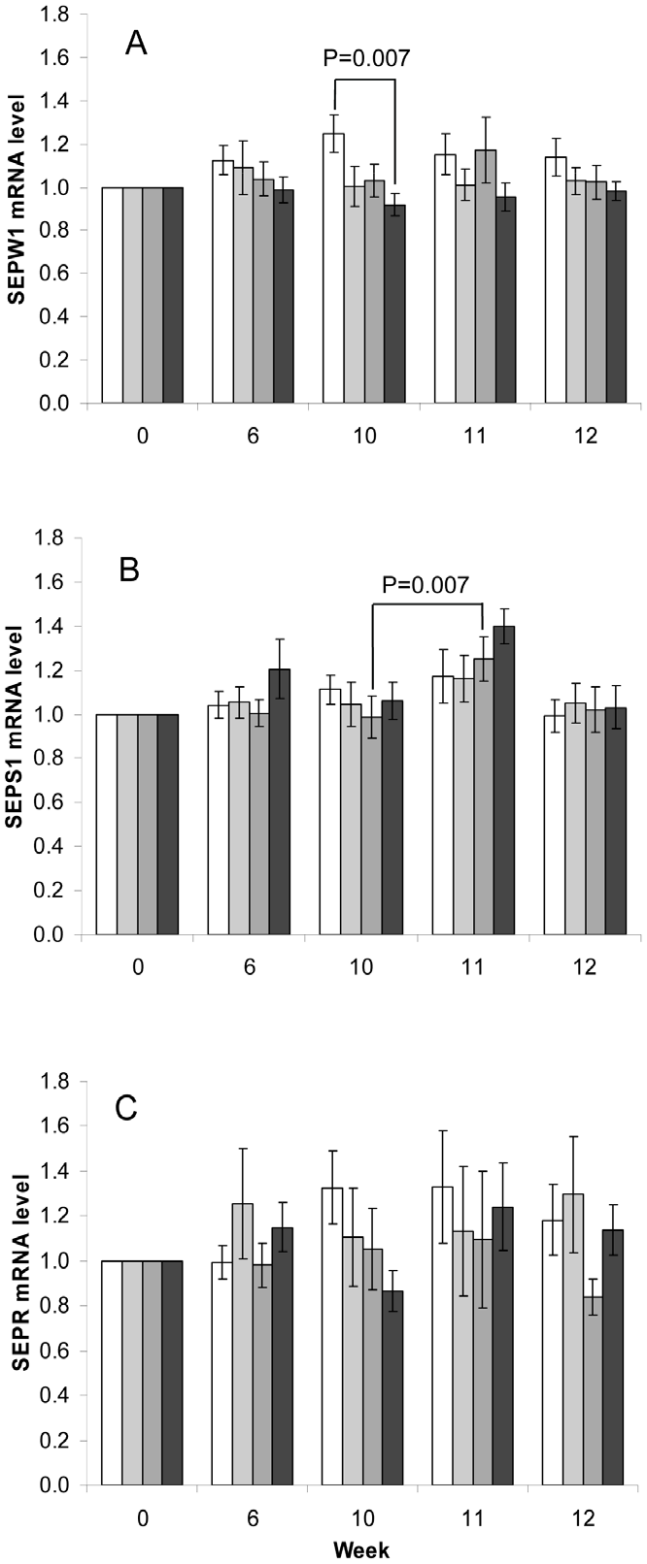
mRNA level in PBMCs measured over the duration of intervention period for (A) SEPW1, (B) SEPS1, (C) SEPR in the placebo and Se-yeast groups. Values are means ± SEM relative to baseline, week 0 expression. GUSB was used as reference gene for normalisation. White bars =  placebo group (n = 14 to 20); light grey bars  =  50 µg/day Se-yeast (n = 11 to 15); mid grey bars =  100 µg/day Se-yeast (n = 10 to 19); black bars  = 200 µg/day Se-yeast (n = 14 to 18). The variation in sample number (n) between time points for each treatment is due to insufficient RNA at some sampling time points and missing time course sample data for some of the target genes. For the SEPS1 gene expression data set: placebo group n = 20 at all time points; 50 µg/day group n = 14 at wks 0, 6 and 12 (n = 13 at wks 10, 11); 100 µg/day group n = 19 at wks 0, 6, 10 (n = 18 at wks 11, 12); 200 µg/day group n = 18 at wks 0, 6, 12 (n = 17 wk 10, n = 15 wk 11). Data were analysed using mixed-effects models and statistically significant differences are indicated on the figure.

A significant treatment effect of Se-enriched yeast on PBMC SEPW1 mRNA level was identified at week 10. The SEPW1 mRNA levels were 25% lower in the 200 µg/day Se-yeast group compared with the placebo group at this time point (p = 0.007) (Figure 2). The SEPW1 mRNA level of the placebo group and the lower dose Se-yeast groups (50 or 100 µg/day) did not change significantly at weeks 6, 10, 11 or 12. The reduction at week 10 in SEPW1 mRNA levels was negatively correlated with plasma Se concentration (P = 0.022); a negative trend was also observed at the majority of the sampling points of the intervention period (Figure 2). No significant differences were found in the levels of SEPR mRNA when comparing the Se-yeast to placebo groups at each sampling point or between supplement groups over the course of the intervention (Figure 2). Inter-individual variation was 40% greater for this marker than for SEPS1 or SEPW1.

**Figure 3:**
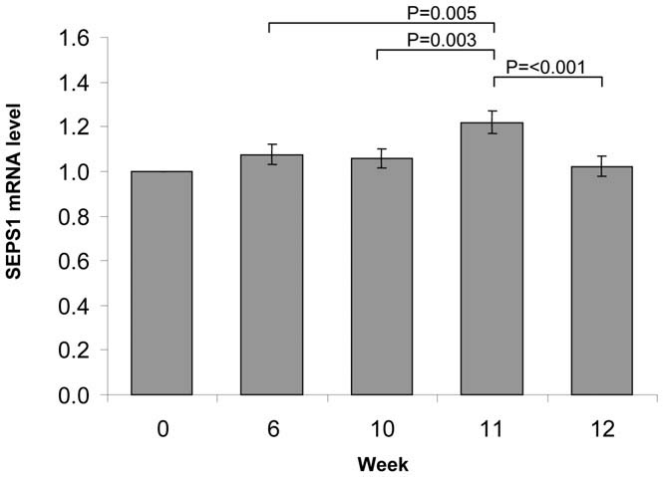
SEPS1 mRNA level in PMBCs measured over the intervention period, each time point showing the mean of all the yeast supplement groups (placebo, 50, 100 and 200 µg/day). GUSB was used as reference gene for normalisation. Data are presented as mean ± SEM (n = 65 to 71 per time point) relative to baseline, week 0 expression.

SEPS1 was significantly up-regulated 7 days after the influenza vaccine challenge at week 10 (P = 0.003) (Figure 3). At Week 11 SEPS1 mRNA levels demonstrated a positive Se dose-dependent correlation (P = 0.009). The SEPS1 mRNA levels in the 200 µg Se-yeast group were on average 16% higher than those of the placebo or 50 µg Se-yeast group and 10% greater than the 100 µg Se-yeast group (Figure 2). A significant effect of time on SEPS1 mRNA level was identified using ANOVA. Post-hoc testing found that SEPS1 mRNA increased (P = 0.007) one week after the influenza vaccine (week 11) compared to pre-vaccination (week 10) in the 100 µg/day Se-yeast group and there was a similar increase in the 200 µg/day Se-yeast group, which was of borderline significance (P = 0.055). SEPS1 mRNA did not change in the placebo group and the 50 µg/day Se-yeast group at week 11 compared to week 10. Two weeks after the influenza vaccination (week 12) SEPS1 mRNA fell to levels comparable with those at week 10 (pre-vaccination).

**Figure 4:**
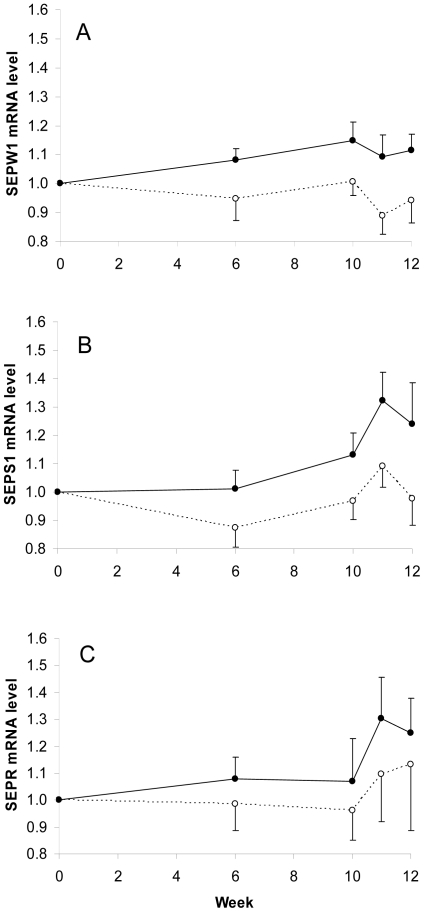
SEPW1, SEPS1 and SEPR mRNA level over the duration of the intervention in the PBMC samples from the unenriched and Se-enriched onion meal groups. SEPW1, SEPS1 and SEPR mRNA levels were quantified using real time RT-PCR and normalised to reference gene, GUSB. Data expressed are means ± SEM relative to baseline, week 0 expression. (A) SEPW1, (B) SEPS1, (C) SEPR. Values are means ± SEM. ---○--- unenriched onions, n = 14 to 16;—•— Se-enriched onions, n = 16 to 17. SEPW1 mRNA level is significantly greater in the enriched onion group compared to the unenriched onion group (p = 0.012). SEPS1 mRNA level is significantly influenced by week (p = 0.009).

When the gene expression profiles of SEPS1, SEPW1 and SEPR in the PBMC samples from volunteers in the Se-enriched onion group are compared with the unenriched onion group, there was a consistent trend with mean mRNA levels of SEPW1, SEPS1 and SEPR being higher in the Se-enriched onion group (Figure 4). There was a significant treatment effect on SEPW1 mRNA, with higher levels in the Se-enriched onion group (P = 0.012) compared to the unenriched onion group. There was also an increase in SEPS1 mRNA levels in the Se-enriched onion group compared with the unenriched onion group, but this was only borderline significant (P = 0.059). SEPS1 mRNA levels were significantly influenced by time (P = 0.009) which was largely due to the increase in expression at week 11, one week post-vaccination. Differences in gene expression of SEPR when comparing the Se-enriched onion group with the unenriched onion group showed a similar pattern of expression to that of SEPS1 but the changes in SEPR over time and comparing treatments were not significant due to the relatively small average fold change in expression between the groups and also due to large inter-individual variation in gene expression/mRNA level.

The main finding of this study was the up-regulation of SEPS1 mRNA in response to influenza vaccine, with a dose-dependent relationship between the magnitude of increase in SEPS1 gene expression and the level of Se supplementation.

## Discussion

The ranges in GPx1 and GPx4 activities in erythrocytes and platelets in this study were similar to values observed in another UK cohort [18]. It has been reported that platelet GPx1 and GPx4 activities reflect Se status more accurately than other blood Se biomarkers [18], [36]-[38], however, the response of GPx1 activity reaches a plateau at approx 80–100 ng/ml plasma Se [10], [12], [36] and so the use of platelet GPx activity as a biomarker of status is restricted to Se- deficient populations. No significant differences in the Se-dependent GPx1 activity in platelets in this cohort were reported previously [24]. In this study total GPx1 activity in platelets (short-term marker of status) was analysed using a different substrate (cumene hydroperoxide) for the enzyme activity quantification [29], compared to the data previously published [24], plus analysis of erythrocyte GPx1 as a long-term marker of Se status [10] was completed. Platelet Se-dependent GPx4 activity was also quantified to ascertain whether a further array of relevant antioxidant enzyme activities in blood cells would reflect Se status in this study population. Although there were significant changes in platelet total GPx1 and Se-dependent GPx4 activities in platelet samples from the 100 µg/day Se-yeast group at week 12 compared to baseline, none of the four Se supplemented groups showed significant increases in total GPx activity over the duration of the intervention compared to the placebo group, most probably because the majority of volunteers had habitual Se intakes associated with maximal GPx activity (the plasma Se concentration at baseline was 95.7±1.5 ng/ml). Total GPx1 activity in platelets and Se-dependent GPx1 activity in erythrocytes were not sensitive biomarkers of Se status within the range of this intervention study. This was also the case with GPx4, as GPx4 activity may plateau over a similar range. Furthermore, the small but not significant increases in GPx4 over time, when compared to baseline week 0, (observed in the 50 and 200 µg/day Se-yeast and Se-enriched onion groups), may be related to the form of Se used in this intervention as sodium selenite supplements of 100 µg /day resulted in a significant increase in GPx4 activity in lymphocytes in another UK cohort [8].

Between 10–30% of Se in plasma is found in GPx [13], [39] and approximately 25–50% in selenoprotein P [13], [39], [40]. There is also a proportion of Se bound to albumin [13], [39], and ‘unknown’ selenoproteins and small Se metabolites account for the remaining plasma Se [13]. Total plasma Se and selenoprotein P concentrations are good markers of Se status [19], [24], but plasma Se does not reflect the intake of all forms of Se [24]. The results presented in this paper show that the steady-state plasma Se concentrations achieved in the different intervention groups were not significantly changed by influenza vaccine administration.

Molecular assays are increasingly used to assess disease and health status and may be useful for the evaluation of nutritional status [41], [42]. A number of studies using animal models have successfully used molecular markers to identify significant differences between groups deprived of dietary Se and those with adequate Se diets [43]-[46]. A comparison of tissue mRNA levels in Se-deficient compared to Se-replete rats reported reductions of both thioredoxin reductase and SEPW1 mRNA by up to 70% in Se deficiency [45], [46]. The *in vitro* expression of SEPW1 mRNA in human colon cells increased by 3.7 fold in cells cultured in media supplemented with sodium selenite compared to media containing sub- optimal Se content [46]. The data presented here do not, however, reflect the magnitude of change in molecular markers of Se status that is observed in animal or *in vitro* models. This is likely due to higher inter-individual variability in human subjects and may also reflect the tight regulation of selenogene and selenoprotein expression. In addition, the level of Se deficiency routinely used in animal studies [45], [46] does not compare directly with the marginal sub-optimal status observed in the volunteers on the study. Furthermore, animal models have different tissue distribution and expression of Se metabolising enzymes and, in particular, rats may not be ideal models to study effects of all forms of Se, in particular monomethylated species [47].

In a longitudinal study of 39 human subjects, Sunde and colleagues found no correlation between mRNA levels of SEPW1, selenoprotein P, selenoprotein H, GPX1, GPX3, GPX4 and plasma Se over 24 weeks [48]. The explanation proposed was that the volunteers were on the plateau of the response curve for these markers, and as such had a replete Se status with respect to expression of the molecular markers measured. However, the average plasma Se concentration was 1.13±0.16 µmol/l [48] whereas the average plasma Se in volunteers recruited on the present study was 1.21±0.13 µmol/l [24]. It is likely therefore that our volunteers were on the plateau of the response curve for SEPW1 and SEPR which would explain why supplementation with additional Se, up to 200 µg/day, did not produce a consistent significant change in gene expression of SEPW1 and SEPR. SEPW1 does, however, present an exception at week 10 where mRNA levels were negatively correlated with Se-yeast dose (up to 200 µg/day) when a steady state Se status was achieved based on plasma Se data [24]. This change in SEPW1 gene expression would not have been encountered by Sunde *et al* [48] as their study focussed on differences in molecular markers over a range of habitual intakes estimated to be 27–83 µg/day and the effect of Se supplementation on SEPW1 gene expression was not investigated [48].

Supplementation with Se-enriched onions demonstrated a consistent, albeit relatively small, increase in the level of mRNA of all the selenoproteins tested, when compared with the unenriched onion group, particularly SEPW1. SEPW1 significantly increased in the Se-enriched onion group compared to the unenriched onion group. This result was as expected from *in vitro* work with Se-methylselenocysteine adapted human cells [49], as the predominant form of Se in onions is γ-glutamyl methylselenocysteine (66%) [24], [50]. In contrast to the Se-enriched onions which contain <9% selenomethionine; the major form of Se in Se-yeast is selenomethionine, constituting ∼60% of the Se content [51]. Supplementation with 200 µg/day L-selenomethionine was shown to up-regulate expression of 28 genes [52] but the individuals selected had arsenic-induced pre-malignant skin lesions and many of the genes found to be up-regulated were involved in immunological and oxidative stress regulation, which would likely have been differentially regulated in individuals suffering from this condition compared to healthy individuals. Additionally, no selenoprotein genes were found to be differentially regulated by the supplementation regimen. The effect of form of Se in Se-enriched onions on expression of key genes encoding selenoproteins, plus expression/activity of important selenoproteins warrants further investigation.

The lack of compelling evidence for the regulation of SEPR and to a lesser extent SEPW1 expression in PMBC in response to Se supplementation, over the range of intakes and time points tested, is likely to be partly due to high inter-individual variation which would mask potentially relatively small changes in mRNA level. The level of inter-individual variation in PBMC gene expression has been found to be inherently high [31]. In a previous intervention study using a dietary supplement of 100 µg sodium selenite/day, the authors were only able to identify changes of 1.2 fold difference between Se supplemented and un-supplemented individuals in ribosomal protein L30 (RPL30), L37A (RPL37A) and eukaryotic translation elongation factor 1 epsilon 1 (EEF1E1) genes [53]. This was attributed to the fact that the main control mechanisms of the targeted genes are predominantly at the post-transcriptional level [53], which may also be the case for the genes we investigated. Furthermore, although work with animal models has identified some highly Se responsive mRNA species the majority of the selenoproteome appears to be unaffected by dietary Se variation [54], [55]. The effects of Se on gene expression may also be form-specific and dose-specific, as highlighted by specific changes in SEPW1 and SEPS1 in response to different treatments in the present study.

A significant increase was observed in SEPS1 mRNA at week 11, one week after influenza vaccine was administered, but it should be noted that one limitation of this study was the lack of a vaccine control group. SEPS1 is known to protect the functional integrity of the endoplasmic reticulum by the removal of misfolded proteins and to modulate cytokine production [23], [56]. The modulation of cytokines is hypothesised to function in a regulatory loop, whereby cytokines elicit increased expression of SEPS1 which then inhibits the production of further cytokines [57]. Our results are the first observation of a Se dose-specific up-regulation in SEPS1 mRNA in response to influenza vaccine, as a marker of immune function effects. The increase in SEPS1 expression in reaction to such a challenge concurs with its hypothesised key role in the regulation of cytokines which control the body's inflammatory response [56].

In conclusion, SEPW1 and SEPR were not sensitive molecular markers of exposure to different forms and levels of Se, and did not significantly change after influenza vaccine challenge in the population studied. However, quantification of mRNA levels of SEPS1 in different Se-supplemented groups after influenza vaccine indicated a dose-specific response in SEPS1 expression after vaccination. This potentially important finding should be investigated further, especially in relation to the potential role of SEPS1 in the immune response.

## Supporting Information

Figure S1 Flow diagram to represent the number of volunteers who were screened and recruited onto the study. Reproduced with permission by Hurst et al 2010 [24].
(0.34 MB TIF)

Text S1 Approval Letter from Norwich Ethics Committee
(0.08 MB DOC)

Protocol S1 Trial Protocol
(0.23 MB DOC)

Checklist S1 CONSORT Checklist
(0.04 MB DOCX)
