# Supplementary figures and images for: Effects of Selenium Supplementation on Selenoprotein Gene Expression and Response to Influenza Vaccine Challenge: A Randomised Controlled Trial
Source: PLoS One. 2011 Mar 21;6(3):e14771. doi: 10.1371/journal.pone.0014771 (PMC3061857; doi:10.1371/journal.pone.0014771)

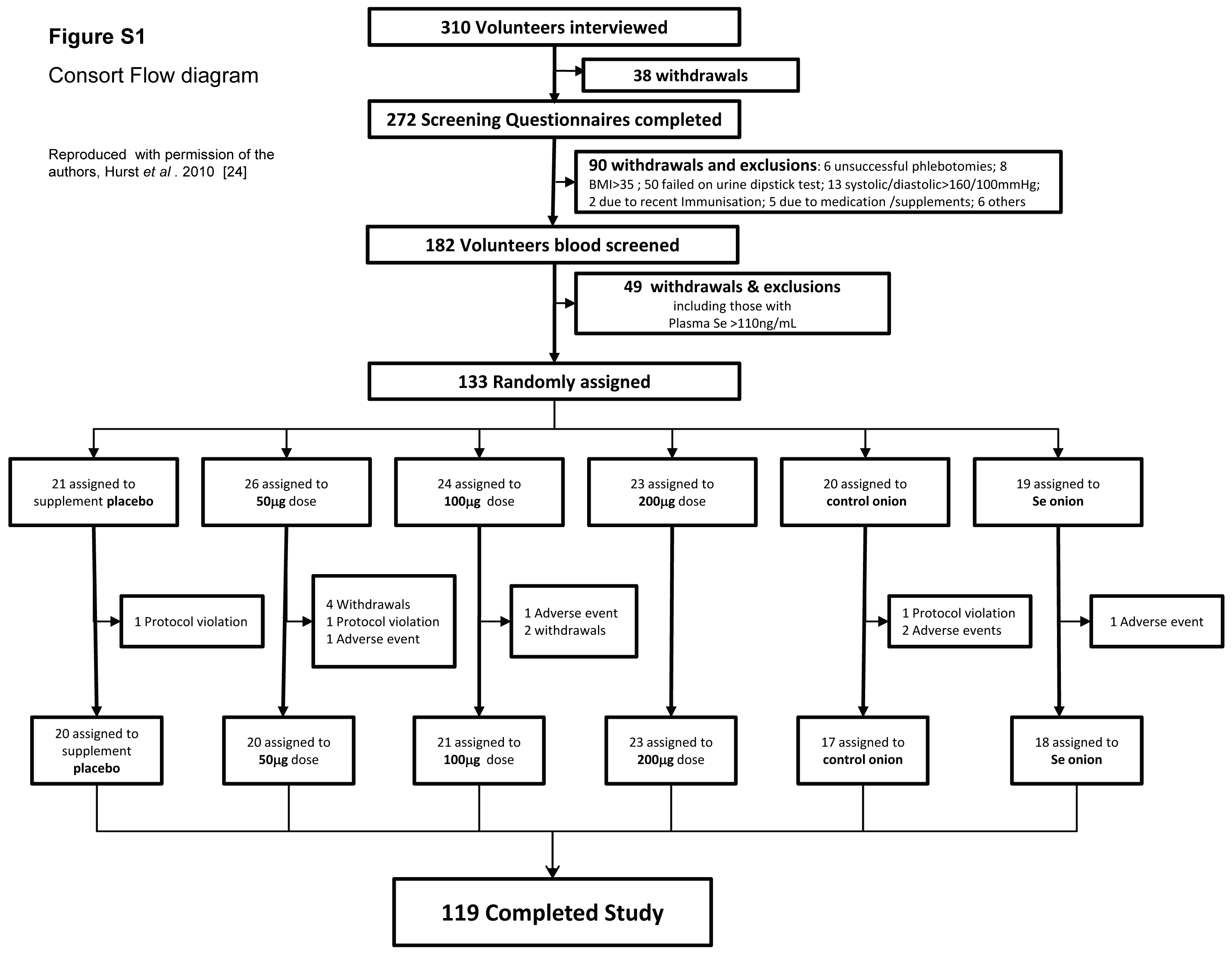

Supplement: Figure S1 — Flow diagram to represent the number of volunteers who were screened and recruited onto the study. Reproduced with permission by Hurst et al 2010 [24]. (0.34 MB TIF) [file pone.0014771.s001.tif]
