# Supplementary material for: Effects of Selenium Supplementation on Selenoprotein Gene Expression and Response to Influenza Vaccine Challenge: A Randomised Controlled Trial
Source: PLoS One. 2011 Mar 21;6(3):e14771. doi: 10.1371/journal.pone.0014771 (PMC3061857; doi:10.1371/journal.pone.0014771)
Supplement: Protocol S1 — Trial Protocol (0.23 MB DOC) [file pone.0014771.s003.doc]

**
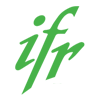
 *Institute of Food Research***

**Selenium Status and Immune Function**

**The Selenium Study**

**Principal Investigator:**

**Prof Susan Fairweather-Tait**

**1.0 Aim**

**2.0 Scientific Background**

**3.0 Objectives**

**4.0 Study Design**

4.1 Overview

Figure 1 Study Outline

**5.0 Recruitment strategy**

5.1 Basic Inclusion criteria

5.2 Basic Exclusion criteria

5.3 Eligibility Assessment

5.4 Eligibility/Clinical Screening

**6.0 Study Protocol**

6.1 Dietary Intervention with Se-yeast

6.2 Dietary Intervention with Onions

Figure 2 Deltoid Muscle

6.3 Sample Collection

6.31 Blood

6.32 ~~Faecal~~

6.33 Urine

6.34 Saliva

6.4 Volunteer Randomisation

Table 1: Study Group Outline

6.5 Supply of Food and Supplements to Volunteers

6.51 Selenium-enriched onions

6.52 Selenium-Supplements

6.53 Compliance

6.6 Sample analysis

6.61 Selenium Determination

6.61i Determination of selenium in Serum (and urine)

6.61ii Determination of selenium in Onions and Meals

6.62 Blood

6.62i Selenoprotein and selenium-biomarkers analysis

Preparation of plasma, erythrocytes and platelets from whole blood

Platelet separation

Erythrocyte and plasma separation

Determination of Protein Concentration

Analysis of GPx1 activity

Analysis of glutathione peroxidase 3 (GPx3) activity

Analysis of GPx4 activity

Analysis of thioredoxin reductase (TrxR) activity

Determination of key selenium-responsive protein levels

RNA isolation from blood samples

Microarray analysis of global gene expression

Analysis of key selenium-responsive genes-RT-PCR (TaqMan analysis)

6.62ii Blood: Cellular and Humoral Immune Response

NK- and CD8-mediated cell cytotoxicity

In vitro analysis of cytokines production

Isotype-specific antibody titres by ELISA

Tissue culture and bi-parametric DNA/BrdU analysis for flu-specific proliferative response

Analysis of expression and distribution of NF-kB

- 1. Faecal Samples
  2. Saliva Samples: Humoral immune response

Detection of individual antibody forming cells by Enzyme linked immunospot (ELISPOT)

Isotype-specific antibody titres by ELISA

6.65 Determination of alpha-tocopherol in serum

**7.0 Statistical Analysis**

**8.0 References**

# 1.0 Aim

The aim of the project is to investigate the relationship between dose and form of selenium and immune function, and to identify functional markers of selenium status.

# 2.0 Scientific Background

Selenium is involved in the maintenance of antioxidant defence systems and immune function. Although approximately 30 selenoproteins have been identified and more than half characterised, none is a universally accepted functional marker for selenium status. At present, dietary requirements for selenium are based on intakes that maximise activity of the selenoenzyme GPx (Standing Committee on the Evaluation of Dietary Reference Intakes, 2000) but there is considerable debate as to the appropriateness of this endpoint. In addition, dietary recommendations are now focussed on optimal health, not just preventing deficiency disorders, which requires a greater understanding of the reported effects of supra-nutritional intakes of selenium, the relative merits of different forms of selenium, and the impact of marginal selenium status on immune function (Clark et al, 1996; Yoshizawa et al, 1998). The consequences of lower selenium status are not entirely clear, however experimental models have shown that marginal deficiency can affect components of the immune system resulting in general immunosuppression and susceptibility to some cancers and viral diseases (McKenzie et al, 1998; Finch & Turner, 1996; Taylor, 1995); Overt selenium deficiency is associated with dilated cardiomyopathy, skeletal muscle myopathy, osteoarthropathy and cretinism in iodine deficient population (Rayman, 1997; 2000).

Dietary selenium is mainly present in organic complexes, such as selenomethionine (SeMet) found in cereals and plant foods, and Se- methylselenocysteine (SeMC) in selenium -accumulator plants such as onions and brassica vegetables, with smaller quantities of inorganic selenium derived from animal-based foods and dietary supplements (Combs, 2001). The metabolism and bioactivity of selenium is dependent on its chemical form: inorganic selenium can be used directly for the synthesis of GPx and other selenoproteins whereas selenomethionine is initially incorporated into tissue proteins, and only released for selenoprotein synthesis after further metabolism to selenide and selenocysteine (Reilly, 1996). The bioactivity of dietary selenium is related to its chemical form; SeMC has been identified as the most potent form of selenium in terms of protection against cancer possibly because, unlike SeMet which is incorporated non-specifically into proteins (Whanger, 2004), it can be converted directly into methyl selenol (Ca et al, 1995), a bioactive substance associated with cancer prevention (Ip & Ganther, 1996).

Recent epidemiological data from a pooled analysis of three clinical trials showed that individuals whose blood selenium values were in the highest quartile had a statistically significant lower risk (OR=0.66) of developing a colorectal adenoma (a pre-cancerous lesion) compared with individuals in the lowest quartile. These studies, therefore, suggest a mechanism for the decreased risk of colon cancer with selenium in which aberrant, age-related CpG island methylation is decreased by the inhibition of DNMT expression and activity. This study gives a unique opportunity to investigate the effect of selenium supplementation using recently developed techniques for the sensitive and accurate quantification of CpG island methylation in exfoliated colon cells present in faeces.

Intakes of selenium in the UK have fallen from a mean of 60μg/day in 1974 to 30-40μg/day in recent years (Ysart et al, 1999) mainly due to the replacement of Canadian wheat (high in selenium) with European wheat (low in selenium); this has resulted in a fall in the mean plasma selenium concentration (Rayman, 1997). One of the proposed consequences of marginal selenium status is impaired immune function. As people age, they are more prone to infections and tumours and this is, at least in part, related to a decline in immune function. One effect of ageing on T lymphocytes is a shift towards a T-helper 2 (Th2) response during an infection, which means a reduced production of Th1 cytokines such as interleukin-2 (IL-2) and interferon-gamma (IFN-γ) (Cakman et al, 1996). In addition, natural killer (NK) cells, responsible for recognising and killing tumour and virally infected cells, exhibit an age-associated decline in activity, particularly in their ability to proliferate and to kill NK-resistant cell lines in response to IL-2 stimulation (Solano et al, 1999). As a direct consequence of this age-related decline of immune effectiveness, infectious diseases remain a significant cause of morbidity and mortality in the elderly. In particular, influenza virus is a globally very important contagion for the elderly (Nicholson et al, 2003) and few other diseases exert such huge toll in terms of suffering, medical consultation, hospital admission and economic loss (CDC-USA, 2003; Thompson et al, 2003).

Selenium supplementation increased antibody titre response to influenza vaccine in elderly individuals (Girodon et al, 1999), whereas other micronutrients (β-carotene, retinol, α-tocopherol and zinc) had no effect on the immune response to influenza vaccine (Gardner et al, 2000). At IFR data has recently been produced indicating beneficial up-regulation of an additional immune function response by selenium (Nicoletti, unpublished). It was initially observed that short-term *in vitro* culture of human PBL with selenium, followed by challenge with influenza virus, dramatically increased the number of CD3^+^ CD8^+^ cytotoxic T cells that contain granzymes and perforins granules. This indicates an increase in cell-mediated immunity which is an important defence against viral attack. Secondly, it was found that there was an up-regulation of IFNα-2, a major player in the anti-viral response. Interestingly, it was also noticed that there was an increase in production of IL-13, important for antibody production (Bajer et al, 2003), by CD3^+^ CD4^+^ T helper cells. Although these results were observed when both selenomethionine and SeMC were used, it seems that the latter is a more potent stimulator of immune response. These data clearly show the potential of selenium to improve both humoral and cellular immunity. Establishing the potential role of selenium as an enhancer of immune response in vivo may provide an evidence-base for public health policy, with important consequences for preventing influenza and similar diseases in the elderly.

The project will consist of a placebo controlled selenium supplementation study and a dietary intervention with un-enriched and selenium enriched onions. In a parallel group design, subjects will be given either one of three doses of Se-yeast (50, 100 or 200μg selenium/day) or a placebo per day or selenium enriched or un-enriched onions (in the form of test meals) for 12 weeks (Figure 1). The study will allow us to investigate the effects of increasing the intake of dietary selenium as SeMet (found in the tablets) or SeMC (which will be the predominant form in the onions). Selenium supplementation studies using selenite have shown that 50 or 100μg/day selenium significantly increased plasma selenium concentration over a period of 6 weeks, at which point a plateau is reached (Broome et al, 2004). However, the intervention will be carried on for longer because we expect the response from Se-yeast to take longer. Outcome measures will include cellular and humoral immune responses to influenza vaccine (looking at the magnitude and the quality of this response; the up regulation of the immune response may or may not be related to viral clearance, as we are using a killed virus, but this will not be addressed in this study), changes in serum/plasma selenium concentration, selected selenoproteins, and expression of selenium -responsive genes and associated proteins. At IFR, we are currently investigating the effect of selenium form and dose on global gene expression in cell culture studies (micro array data), and have identified several selenium -responsive genes; quantitative RT-PCR (Taqman) analysis of mRNA from one gene (selenoprotein W1) demonstrated a linear dose-response within the physiological range of selenium (Hurst, unpublished). Results from our cell culture studies will indicate which genes and proteins to investigate in the human intervention study.

# 3.0 Objectives

- To examine the effect of two different organic forms of selenium that are naturally found in the diet, one of which (SeMC) is purported to confer protection against cancer.
- To provide evidence for a beneficial effect of increasing selenium intake in adults with regard to resistance to influenza virus.
- To investigate the regulation of selenium-responsive genes and protein expression in response to dietary selenium.

# 4.0 Study Design

## 4.1 Overview

A 12 week intervention will be carried out in which 6 groups of 50-64y old men and women (n=24 per group) with low plasma (or serum) selenium are fed one of three doses of selenium (50, 100 or 200µg/d) as selenium enriched yeast (Se-yeast) tablet, a placebo tablet, selenium-enriched onions (equivalent to 50µg/day selenium) or un-enriched onions during the summer, autumn and winter periods (July-February). Subjects will be given an influenza viral challenge 10 weeks after starting the intervention. Volunteers will be informed that our study also vaccinates during the months December to February which is outside the traditional flu vaccination period adopted by the NHS. The precise time of vaccination for the volunteers will depend on their intervention starting date and may therefore take place in the period between December to February. Health care workers and those who are offered free flu vaccination from the NHS will not be eligible to take part in the study during the months of December to February.

The Se-yeast treatment will take place over 3 years; the onion treatment will take place during years 2 and 3 (the onions will be grown in years 1 and 2 for consumption the following year). The onions will be stored for a number of months and then incorporated into meals which will be stored at -18°C until required.

The control subjects will take a placebo and undergo the same tests. Venous blood samples will be taken pre-vaccination at baseline (week 0, 80mls), 6 (40mls) and 10 weeks (80mls) then post vaccination at 11 (80mls) and 12 weeks (80mls). Saliva samples will be obtained at baseline (pre-vaccination) and 12 weeks (post-vaccination). Blood samples will be used for the assessment of plasma selenium concentration (Q-ICPMS), plasma vitamin E (HPLC), selected selenoproteins, selenium-responsive genes, protein expression, and cellular and humoral immune response. The saliva samples will be tested for antibody titres to influenza antigens. A 24hour urine collection will be made during week 6 to determine selenium concentration.

Week 0

6

10

11

12


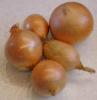


Up to 5 onion based test meals consumed

per week for 12 weeks


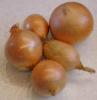


-Se

+Se

1 capsule taken every day for 12 weeks

Fasted Blood sample (60mL)

24hour urine collection

Influenza virus vaccination

Volunteer groups taking part in study

Saliva sample

**Fig 1: Study outline**

Vaccination with influenza virus will be given during week 10 of the intervention and blood samples (80mL) taken 7 and 14 days post vaccination. These will be used to determine selenium supplementation-induced changes in mucosal and systemic humoral and cellular immune responses.

# 5.0 Recruitment Strategy

The study population will consist of six groups of apparently healthy 50-64y old men and women (n=24 per group) with low plasma (or serum) selenium. The volunteers will be recruited until 144 have completed the study. Experience has shown that the drop out rate for these types of study is 25%. If a greater drop out rate occurs recruitment will be reviewed.

Advertisements (annex 3) will be placed around the Norwich Research Park (University of East Anglia (UEA), John Innes Centre (JIC), and IFR, in the HNU news letter (which is sent to all volunteers on the HNU volunteer database) in the local press and other suitable locations for example, supermarkets, social clubs, church news letters and golf clubs, gymnasiums and leisure facilities within the local area (40 mile radius). As in the past we will also obtain radio coverage and place advertisements (Annex 17 and 18) in the press to raise local awareness of the project if need be. The posters advertising this study will also be supplied with business cards or other suitable appendages to the poster in order to facilitate volunteer recruitment.

Apparently healthy volunteers from the HNU volunteer database, who meet the basic inclusion criteria, will be sent a letter of invitation (annex 2) to participate in the study. This will be supported by the volunteer information sheet (annex 4). Included will be a response slip and pre-paid envelope for returning the slip if they are interested and wish for further information. The HNU database has approximately 383 people between the ages of 50 and 64 years who are willing to participate in dietary intervention studies. It is known that at least 278 of these are non-smokers, and contact will made with these individuals.

Advertisements will also be placed on appropriate noticeboards in the Norfolk & Norwich University Hospital and on the NNUH intranet. Approaches will be made to GP practices via the Primary Care Research Network. In this instance, the GP will send out letters of invitation to individuals who meet the basic inclusion criteria. This will be supported by the volunteer information sheet and a response slip and pre-paid envelope for returning the slip directly to IFR if they are interested and wish for further information.

### 5.1 Basic Inclusion Criteria

- Men and women, age 50-64,
- Plasma selenium level is <1.2µmol/L (<95µg/L)

### 5.2 Basic Exclusion Criteria

- Elevated blood pressure measurements (<90/50 or <95/50 if symptomatic or >160/100).
- BMI <18.5 or >35
- Results of the clinical screening which are judged by the HNU Medical advisor to be indicative of a health problem and could compromise the well-being of the volunteer if they participated, or which would affect the data.
- Smokers
- Diagnosed with Gastrointestinal disease (excluding hiatus hernia unless symptomatic or study intervention/procedure is contraindicated) for which they have been taking prescription drugs on a chronic basis.
- Diagnosed with a long-term illness requiring active treatment, e.g. diabetes, cancer, cardiovascular disease.
- On regularly prescribed medication known to have a profound effect on the immune function
- Regularly using antacids and laxatives (at least once a week)
- sufferers of Hay-fever taking regular steroid medication
- Unwillingness to discontinue dietary (other than vitamins and minerals) or herbal supplements less than one month prior to the start of the study and for the duration of the study
- Blood donation within 16 weeks of the first study sample and who intend to donate blood less than 16 weeks after the last study sample
- Antibiotic use within four weeks prior to starting the study
- Those who receive or plan to receive any other type of immunisation during the study period
- Those who have received an immunisation within 6months of the start of the study
- Intention to go on holiday/trips for more than 2 weeks during the twelve week intervention
- Those planning a holiday/trip that requires immunisation during the twelve week intervention period
- Parallel participation in another research project which involves dietary intervention or sampling of biological fluids/materials
- Allergic to eggs or egg products
- Allergic to chicken protein
- Allergic to the antibiotic Gentamicin
- A history of Guillain-Barré syndrome

(There is a one in **one million chance (0.000001%)** of volunteers developing the Guillain Barré syndrome on administration of the flu vaccine. It is an acute disease of peripheral nervous system in which the nerves in the arms and legs become inflamed and stop working).

- Those who are offered a free flu vaccination because of a particular medical condition
- Healthcare workers who would receive a free NHS vaccination if their study vaccination would fall outside the NHS normal period (July-December)

### 5.3 Eligibility Assessment

Following an expression of interest volunteers will be invited to the Human Nutrition Unit (HNU) for a preliminary interview and given further details of the study.

The volunteers will be encouraged to ask questions at this point prior to making any commitment. At the end of the interview all volunteers will be given a minimum of 72 hours *(*3 days) to consider whether they wish to participate in the study. They will be given a small sterile container to take away with them. If they wish to participate, this container will be used for the urine sample on the morning of the clinical screening visit. If they decide they do not wish to take part in the study they will be told to discard the container. During this consideration period the volunteers will not be contacted. If, following this period of consideration, the volunteer still wishes to participate they will be asked to contact Dr Charlotte N Armah 255360 or Dr Andrew Goldson 255204*.* If the volunteers are very keen to take part in the study, a date for their screening can be booked at the interview stage. The date agreed upon will be after the 72hours (3 days) consideration time has elapsed.

## 5.4 Clinical/Eligibility screening

All those responding positively will be invited to attend the HNU for a clinical screening following an overnight fast (8 hours*)*. Volunteers will be advised that they should drink water during this fasting period. They will be reminded to bring a midstream sample of urine from the first urine of the morning in the container provided at the first interview. Volunteers need to arrive within 2 hours after collection of the urine sample as this is a required specification for the validity of the urine dipstick test. The urine samples will not be tested until after the consent forms (annex 5+5a) have been signed. As there is an option for the volunteer to take part in the faecal collection, there will be a separate consent form for this.

On arrival at the HNU the study scientist will go through the consent forms with the volunteer and encourage any questions they may have at this stage. Due to the optional nature of one part of the study (the collection of faecal samples to examine the effects of selenium on DNA methylation) the volunteers agreeing to take part in this will then be asked to sign an extra consent form. A medication declaration form (annex 6) agreeing to inform the study scientist of any medication they may have to take will also be signed. A copy of these forms will be given to the volunteer to keep. A qualified nurse will then complete a screening questionnaire (annex 7) take and record blood pressure, pulse, height and weight measurements, calculate Body Mass Index (BMI), perform the urine dipstick test (Combur ^9 Test^®, Roche Diagnostics Ltd). An 8mL blood sample will be taken for Full Blood Count (FBC) and glucose analysis. The blood sample will be sent to the BUPA hospital for analysis. A further 7mL of blood will be used for serum (or plasma) selenium determination which is the major part of the screening process. The analysis of the serum samples will take place on a weekly basis at the end of a week of screenings. The system of analysis can be run whether two volunteers are screened in one week, or twenty. The results from the analysis will usually be known on the day and so the volunteers will not be kept waiting to hear if they have passed the study or not. The volunteers will be made aware of the fact that even though their blood results may be within range, they may be excluded from the study due to inappropriate selenium status.

The urine results (annex 8) will be known immediately. If any of the results for the urine dipstick test are outside the reference range the nurses will refer to the Protocol for Abnormal Urinalysis Results which provides advice on exclusion or re-screening of the volunteer. This protocol has been authorised by the HNU Medical Advisor.

If the BMI is <18.5 or >35 the volunteer will be excluded from the study. If any of the results for the screening blood tests fall outside the standard reference ranges they will be assessed by the HNU Medical Advisor as to whether the results could affect the study data or have implications for the health of the volunteer, and therefore whether inclusion, re-screen or exclusion is appropriate. The HNU Medical Advisor may say that even though results are outside the reference ranges, either a re-screen or inclusion may be appropriate, since some minor deviations will not warrant a re-screen and will neither affect the study data nor have health implications for the volunteer.

If a re-screen is considered appropriate the volunteer will be given the option of returning for another blood test after a period of two weeks. Volunteers who do not wish to be re-screened or who display screening parameters outside the standard reference ranges on both occasions will be excluded from the study.

Copies of all clinical results will be sent to the volunteer’s GP (a letter to GP accompanying clinical results that have passed the screening-Annex 10) and in the event of flagged results (a letter to GP accompanying clinical results that have not passed the screening-Annex 11) the volunteer will be notified by letter (letter to volunteer informing them that they have not passed the screening and that they should contact their GP-Annex 12) advising them to speak to their GP to discuss the results. Results will not be discussed with the volunteer.

Volunteers who meet the study criteria and whose screening results are satisfactory will be contacted and offered appointments at the HNU to continue with the study. The GPs of those successfully recruited onto the study will be informed of their patient’s participation in the study by letter (annex 9) and will be sent copies of all clinical results. As the study involves the administration of the flu vaccine, the letter will also ask the volunteers GP if there is any reason why the volunteer should not be having the vaccine. The GPs will be asked to contact the study team if there is any contraindication. The volunteer will agree to this information being sent to the GP by signing the consent form(s) (Annex 5 and/or 5a). If there is any contraindication, the volunteer will not be allowed to continue with the study and will therefore be excluded. A letter would then be sent to the volunteer explaining this (Annex13). Once the flu vaccine has been administered a copy of the vaccination record, completed by the HNU research nurses, will be sent to the GP.

Once recruited onto the study, volunteers will be assigned a code. The information linking codes to volunteers will have restricted access. All personal information will be kept confidential and known only to the Principal Researcher and project leader, HNU research nurses, the volunteer’s GP and the HNU medical advisor.

## 6.0 Study Protocol

## Due to the nature of the study (onions only available in year 2 and 3) and the fact that some volunteers may have a dislike or intolerance to onions it would be difficult to fully randomise the study and unethical to accept those volunteers onto the onion part of the study. In year 2 and 3, which includes the onion, intervention, volunteers with no dislike of onions will be randomly allocated to one of six groups.  Volunteers with a dislike of onions will be randomly allocated to one of four groups.  There is no evidence to suggest that volunteers who do not or cannot eat onions will display any differences in immune function response or selenium status to those volunteers that do, and thus there will be no consequences of the study not being fully randomised.  However, as this is a new area of research, the preferences of all subjects recruited in the first year of the study will be monitored, and the possibility of differences by onion “preferences” investigated.

## Volunteers will be stratified by sex.

## 6.1 Supplementation Intervention with Se-yeast

Se-yeast supplementation (groups 1-4) will be studied during years 1, 2 and 3 of the project. All the pressed tablets will be identical (bar alphabetical labelling which will be carried out at manufacture) and will be labelled according to the law. Safety advice will also be given when the tablets are handed to the volunteers. One tablet will be taken each day for 12 weeks. The volunteers will also be given a record sheet where they will be asked to indicate at which times the supplement was taken each day (Annex 16).

Group 1: Se-yeast tablet (50μg Selenium/day)

Group 2: Se-yeast tablet (100μg Selenium /day)

Group 3: Se-yeast tablet (200μg Selenium /day)

Group 4: Placebo (control group).

On Day 1 of the study, the volunteer will be asked to attend the unit in the morning between the hours of 7:30 and 9:00am to give their first fasting blood and saliva sample. A scientist from the study team will be on hand to ensure that the volunteer is relaxed and comfortable. The volunteers will be asked not to brush or floss their teeth prior to arriving at the Unit as the use of toothpaste may interfere with the saliva sample to be collected. The volunteer will however be encouraged to bring their toothbrush and tooth paste with them to the unit so they may brush their teeth at an appropriate time, when all samples have been taken. All phlebotomy will be carried out by a qualified nurse at the HNU. A baseline venous sample of 80mL will be obtained for later determination of full blood count and glucose, as well as plasma (or serum) selenium levels, selected selenoenzyme activity, key selenium-responsive genes and protein levels and immune function markers**.** A saliva sample will also be collected from the volunteers after the blood has been taken, so the volunteer can stimulate their saliva glands by chewing sugar free gum while their blood is being taken. The volunteer will be asked to rinse their mouth out twice with cool water before chewing the gum and subsequently producing the saliva sample. The saliva samples will be used to test for a baseline of antibody titres to influenza antigens.

After the consumption of a standard unit breakfast (a range of cereals, toasted bread, tea or coffee), the volunteer will be supplied with a 6 week supply of their Se-yeast tablets and a container for carrying out a 24hour urine collection prior to their next visit in week six. The volunteer will not know what dose of selenium they will have been given neither will the scientist (double blind). The volunteer will be advised to take one tablet everyday for the next 6 weeks, preferably with meals. The volunteer will be given a record sheet to indicate at which time of each day they are taking their supplement. The volunteer will be advised to contact either Charlotte Armah (01603 255 360) or Andrew Goldson (01603 255 204) if they have any queries or problems associated with the Se-yeast tablets.


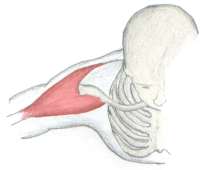
Prior to the start of week six (Day 42±2days), the volunteer will be contacted by telephone or e-mail and reminded when to start their 24hour urine collection, which they will bring with them when invited to the Institute again in the morning between the hours of 7:30 and 9:00am for their second fasted blood sample. No saliva sample will be collected in week 6.

**Figure 2: The deltoid muscle**

The volunteer will again be given a standard breakfast. The volunteer

will then be given their second batch of Se-yeast tablets which will cover their supplementation until the end of the study (week 12).

At the start of week ten, (day 70±2days), the volunteers will come in for their 3^rd^ fasted blood sample in the morning between the hours of 7:30 and 9:00am. The blood sample (80mL) will be taken and before the volunteer is given their standard unit breakfast. They will be given their flu vaccine at the HNU by the qualified nursing staff. The preferred site of administration in adults is the deltoid muscle.

Please note that volunteers will be asked to inform us if they suffer from a cold at the time of the scheduled flu vaccination. If this is the case then we will re-schedule the vaccination appointment. Volunteers will be asked to continue taking the supplements or onion meals as before. In the unlikely event that a volunteer feels unwell for more than 2 weeks we will have to exclude him/her from further study participation.

After the administration of the flu vaccine, the volunteer must remain in the HNU for at least 20minutes and preferable consume breakfast. This is to ensure that there is no anaphylactic response following vaccination. After the standard unit breakfast has been consumed the volunteers will continue to take the Se-yeast capsules as per usual. The volunteer will return to the unit to give fasted blood samples 7 (week 11) and 14 (week 12) days post vaccination (80mL each time); a saliva sample in week 12 and again the volunteer will have the option of producing a faecal sample in week 12. The same procedure will be followed for the collection of the faecal sample in week 12 as prior to week 1. All samples produced until 7pm will be collected from the volunteer’s home by a member of the study team. Volunteers who produce samples after this time will be asked not to collect. The completion of a 12 week Se-supplementation period will mark the end of the volunteer’s participation in the study.

Uncertainty about delivery dates for flu vaccines cause logistical problems. We cannot delay the supplement/dietary intervention until we have confirmation of the delivery date of the flu vaccine. We also need to achieve a continuous flux of volunteers as we are limited to the number of immune function assays we can manage per week. In each project year the first volunteers start the intervention in July so that week 10 (vaccination) falls into the second half of September a date that was originally be considered feasible for the flu vaccine to be available after having contacted several suppliers. However, a delay in flu vaccine deliveries is a possibility and our suggestion is to allow for some flexibility if this happens. We suggest that volunteers that reach week 10 may continue with supplement/onion meals for a maximum of 3 more weeks if the flu vaccine is not available. If the flu vaccine is still not available at week 13 then a final blood sample will be taken for biomarker and immune function analysis. This approach still allows us to complete our measurements of selenium biomarkers but is less likely to show any effect with respect to immune function. However, this should only affect a limited number of volunteers. How many is difficult to predict as we don’t know for some time when the flu vaccine will be available. We feel that this approach is an acceptable solution as we will still meet all the objectives related to plasma selenium status and biomarkers of selenium status.

**Further, we also suggest extending the current flu vaccination period (September to February) to include March and April 2007 and 2008. We will always endeavour to not vaccinate in March and April but we are dependent on the actual flu vaccine delivery for the life time of this project. The HRGC protocol (Annex 1) and volunteer information (Annex 4) have been amended accordingly.**

**6.2 Dietary Intervention with selenium-enriched onions**

Selenium-enriched and un-enriched onions (groups 5 and 6) will only be studied during years 2 and 3 (the onions will be grown in year 1 and 2 of the project). The possible side effects to eating any onions are bloating and wind. This will be clearly stated in the volunteer information sheet (annex 4).

Selenium-enriched onions (in the form of 3 to a maximum of 5 meals per week), providing the equivalent of 50μg selenium/day.

Un-enriched onions (in the form of 3 to a maximum of 5 meals per week), providing <1μg selenium per day.

On Day 1 of the study, the volunteer will be asked to attend the unit in the morning between the hours of 7:30 and 9:00am to give their first blood and saliva sample all subsequent procedures will be the same as those outline in section 6.1. The only difference will be that after the consumption of a standard unit breakfast (a range of cereals, toasted bread, tea and coffee), the volunteer will be supplied with up to one months supply of their onion test meals in a container that will ensure that the meals remain frozen (cool box or cool bag). This will be assessed on an individual basis as the volunteer may not be able to transfer the food in a suitably frozen state to their freezer at home, or may not in fact be going directly home after their visit to the HNU resulting in the thawing of the meal. If this is the case, a food delivery will be arranged (by refrigerated van, hired for this purpose) prior to their commencement of the study (Prior to day 1). The volunteer will also be given a container for carrying out a 24hour urine collection prior to their next visit in week six. The volunteer will not know whether their test meals contain the selenium enriched onions or the un-enriched onions. This will have been randomly allocated on recruitment. The research scientist in charge of the study will also not know, but the allocation of the onions meals will be supervised by a scientist not on the study. The volunteer will be asked to eat up to 5 of the onion test meals each week for the next 12 weeks, returning to their normal diets in between onion meals. The volunteer will be given a record sheet to indicate at which time of each day they are consuming their onion meal (Annex 16).

Arrangements will be made with the volunteer for suitable times for the remainder of their meals to be delivered either by refrigerated van or by a member of the study team; alternatively volunteers will be offered the option to pick up the meals themselves from the Human Nutrition Unit at their own convenience. The volunteer will be advised to contact Charlotte Armah (01603 255 360) or Andrew Goldson (01603 255 204) if they have any problems or queries associated with the consumption of the onion test meals.

The same procedure will be followed in week six (day 42±2days) as outlined in section 6.1 for the supplementation intervention, the only exception being that the volunteer may well be given another batch of meals, or arrangement will have been made for the delivery of test meals.

The same procedure will be followed in week ten as outlined in section 6.1, the only exception being that after the administration of the flu vaccine, the volunteer will continue to consume their thrice weekly onion based test meals as per usual. The volunteer will return to the unit to give fasted blood samples 7 (week 11) and 14 (week 12) days post vaccination (80mL each time) and a saliva sample in week 12.

## 6.3 Sample Collection

### 6.31 Blood Samples

A total of six blood samples (including one taken at screening) will be taken throughout the course of the study, a total of 375mL of blood. On the day of the clinical screening, 15mL of whole blood will be taken. A volume of 8mL will be sent to BUPA for Full blood count and glucose. The other 7mL (in selenium free tubes) will be used to separate serum/plasma for the determination of the volunteer’s selenium levels. The serum/plasma samples will be labelled and stored at -80^o^C until analysis.

On the study days, 80mL of blood will be taken. A volume of 3mL will be sent to BUPA for a Full blood count. 10mL of whole blood will be used for the determination of serum/plasma selenium and alpha-tocopherol levels; 15mL of whole blood fractionated by centrifugation to produce platelets, serum/plasma and erythrocytes to look at the activities of selenoproteins and protein expression levels. A further 12mL will be used for RNA extractions and functional biomarkers of selenium status. 40mL will be required for use cellular and humoral immune response analysis (this will NOT be taken in only week 6).

### 6.33 Urine Samples

The procedure for collection of the sample will be explained to the volunteers. Researchers at the Institute of Food research have many years of experience in collecting urine samples from volunteers and are able to instruct volunteers to collect in a safe and hygienic manner. The urine samples will be brought in by the volunteers themselves when they arrive for their 2^nd^ fasted blood sample in week 6.

### 6.34 Saliva samples

Saliva samples will be expectorated into clean plastic containers after blood samples have been taken in weeks 0 and week12. The volunteers will be advised not to brush or floss their teeth on the morning they are due to come in to give blood. The volunteers will then be asked to rinse their mouths out, at least twice, with cool water. In order to stimulate the production of saliva, the volunteer may be asked to chew sugar free gum for 1-2 minutes. After the time has elapsed, the volunteer will be asked to expectorate into an already labelled sterile wide necked tube (Universal), trying to avoid touching the mouth of the tubes with their hands. The saliva samples will be stored at -80^o^C until required.

## 6.4 Volunteer Randomisation

Ideally the study would be completely randomised, but this would compromise our ability to complete recruitment in the time allowed i.e. only accepting volunteers onto the study that like onions so that they could be randomised into any one of six groups (years 2 and 3 only as year one is the supplement study only). It would also be unethical to accept volunteers who had a dislike or intolerance to onions onto the onion part of the study.  Volunteers will as far as possible be randomly allocated to one of four groups in year one (0µg/day (placebo), 50µg/day, 100µg/day and 200µg/day of Se-yeast) and one of six groups in years 2 and 3 (the selenium supplements above as well as selenium-enriched and un-enriched onion groups).  There is no evidence to suggest that volunteers who do not or cannot eat onions will display any differences in immune function response or selenium status to those volunteers that do, and thus there will be no consequences of the study not being fully randomised.  However, as this is a new area of research, the preferences of all subjects recruited in the first year of the study will be monitored, and the possibility of differences by onion “preferences” investigated. Volunteers will be stratified by sex. The volunteer information document (annex 4) will clearly state that volunteers recruited in 2005 will be assigned to the supplement study only.

|  | Volunteers to be recruited | | |
| --- | --- | --- | --- |
|  | Selenium-enriched onions | Un-enriched onions | Selenium-Supplementation |
| Year 1 (2005-2006) | n=0 | n=0 | n=32 |
| Year 2 (2006-2007) | n=12 | n=12 | n=32 |
| Year 3 (2007-2008) | n=12 | n=12 | n=32 |

Table 1: Study groups outlined

## 6.5 Supply of Food and Supplements to Volunteers

**6.51 Selenium-enriched onions**

The selenium-enriched and un-enriched onions will be grown by the Division of Plant Science, University of Nottingham, under the supervision of Dr Martin Broadley, who is an authority on mineral nutrition of crops and is currently supervising a BBSRC CASE studentship programme on selenium enhancement with Hydro-Agri UK. The UK’s most popular variety of main crop onion (*Allium cepa* L., cv. Renate; a Rijnsburger type) will be grown by an experienced team of specialist horticultural scientists using standard commercial UK horticultural practices, and with the expertise in selenium fertilisation gained from the BBSRC CASE studentship. The onion crops will be harvested in August 2005 and 2006, and stored to ensure quality material is delivered for the intervention study. The selenium content of onions is approximately 1μg/100g (i.e. 0.9μg/onion) whereas Se-enriched onions contain 70-90μg/100g (i.e. 60-80μg/onion). Sufficient onions (~3,000) will be grown to provide enough onions for up to five meals per week for each volunteer for the 12 week intervention (Se-enriched: up to 5(meals) x12(weeks) x24(subjects); un-enriched: 5(onions) x12(weeks) x24(subjects)).

We will request that the different onions are labelled with different coloured tags (for example, or put in different coloured sacks). Meals will then be prepared with say, the red labelled onions, or the blue labelled onions without any one in the kitchen or the leading scientist knowing whether the red or blue labels related to enriched or un-enriched onions. As well as asking the manufactures to keep records of the coding i.e. red for selenium-enriched and blue for un-enriched say, we would ask that we too had an envelope with the information. This information would be held by a scientist not associated with the project, with the information being disclosed at the end of the study.

The volunteers taking part in the study will be given up to 5 meals with the same colour coding each week out of a minimum choice of 5 meals. All test meals will be prepared in a dedicated food preparation kitchen by the HNU diet cooks or in their absence by a member of the study team in possession of the Foundation Certificate in Food Hygiene, level 1. These dishes may include a beef carbonarde (beef in beer), three cheese pasta, ratatouille, shepherds pie and spaghetti bolognaise (sauce). The meals will be made with the enriched and the un-enriched onions and neither the scientist leading the study, nor the volunteer will know which type of onion they are consuming. Each set of meals i.e. the selenium-enriched and the un-enriched will be labelled by colour, for example all un-enriched meals labelled blue and all enriched meals labelled red.

The meals will be delivered to the homes of the volunteer once the volunteer’s capacity to store the food has been assessed, which will usually occur at the initial interview stage. For example if a volunteer has a large freezer compartment, then up to one month worth of food may be delivered in one go. If however the volunteer only has a small freezer box, then it may be more appropriate to deliver food on a weekly basis. All deliveries of food will be made either by refrigerated van or by a member of the study team; alternatively volunteers will be offered the option to pick up the meals themselves from the Human Nutrition Unit at their own convenience. No extra meals will be provided for members of the family and spouses of the volunteers as the cost are prohibitive. The volunteers will be reminded that possible side effects to eating any onions are bloating and wind.

**6.52 Selenium Supplements**

The supplements and the placebos will be produced and supplied by a company called PharmaNord (Denmark) who will make the pressed tablet containing high selenium-yeast. The placebo tablet will be visually identical to the doses but will contain yeast containing no selenium. The supplements used in this study will be from the same batch as those to be used in a human study being carried out at the University of Liverpool, due to start next year. The supplements will be supplied blind to the Institute and labelled either A, B, C or D. The possibility of assessment bias will be avoided by using a double blind assessment. The volunteers will receive their supplements in a blister pack labelled either, A, B, C or D. The supplements will be taken everyday with breakfast from the day of the first blood sample (Day 0). The volunteers will be asked to keep track of when they take their supplement each day by ticking the appropriate box on the record sheet provided. We will request that the manufacturers of the tablets maintain a copy of the code for the supplements and that a copy of the code be sent to us to be held by Aliceon Blair (HNU manager) in the case of a serious adverse event where we would need to know the dose being taken by the volunteer.

**6.53 Compliance**

Meals will be provided and frequent contact with volunteers maintained during the intervention to check consumption of meals. The volunteers will be encouraged to inform us if they are not consuming the whole test meal. Volunteers will be given slightly more tablets than they will need in the blister packs. When the volunteers are invited back for their fasted blood in week six, they will be encouraged to bring the remainder of their blister pack so that we can issue a new set for the next six weeks and count the number of capsules they have consumed from the first set. A 24h urine sample will be collected midway through the intervention as selenium excretion is a good indicator of selenium intake. Serum/plasma selenium concentration will also be used as a check on compliance treatment.

## 6.6 Sample Analysis

### 6.61 Selenium Determination

Selenium determination will be carried out “in house” on an Agilent 7500C ICP/MS. This instrument is fitted with an octapole collision cell enabling elimination of interferences caused by argon dimmers around mass 80. Serum/plasma samples for total selenium status will be collected over a period of one week (one batch) and the analysis will be carried out at the end of that week. Depending on the number of volunteers screened in a particular week, the number of serum (or plasma) samples per batch could vary.

**6.61i Determination of Selenium in Serum/plasma (and urine).**

Selenium in serum/plasma (and urine) samples will be measured following the method of Delves and Sieniawska (1997), with refinement and improvement where possible. This will involve a simple 1 in 15 dilution of 200μL volumes of serum (or plasma) with a diluent containing 1.0% v/v butan-1-ol; 0.66% Triton X-100; 10mMol dm^-3^ NH_3_, 0.2mMol dm^-3^ (NH4)_2_H_2_ EDTA and 2 mMoldm^-3^ NH_4_H_2_PO_4_. This enables the ICP-MS to provide accurate and precise measurements of total selenium concentrations. Interferences from argon-adduct ions are further reduced by the presence of butan-1-ol in the diluent.

**6.61ii Determination of Selenium in onions and meals.**

Determination of total selenium in onions and the test-meals will follow a method described by Shah *et al* (2004), involving the digestion of the sample with nitric acid and hydrogen peroxide. The basic method may include refinements and improvements where possible. Quantification will be carried out by standard addition.

A separate project will be looking at selenium speciation Gammelgaard *et al* (2002) using ion pairing HPLC/ICP/MS.

### 6.62 Blood Samples

**6.62i Selenoprotein and Se-biomarkers analysis**

**Preparation of plasma, erythrocytes and platelets from whole blood**

Blood samples will be fractionated by centrifugation and samples of platelets, erythrocytes and plasma will be prepared as described previously (Fox, 2002).

**Platelet separation**

Whole blood samples will be collected and transferred to an EDTA containing Vacutainer TM tube (Becton Dickinson) and kept at room temperature for no longer than 30 minutes before centrifugation at 10g for 17 minutes to separate the erythrocytes from the plasma. The plasma layer containing the platelets will be transferred to a 1.5mL cryovial for a second centrifugation step at 900g for 12 minutes (room temperature) to separate the platelets. The plasma will then be removed by aspiration and the platelet pellet will be washed twice with modified Hanks buffer pH7.3 followed by centrifugation at 900g for 12 minutes at 4^o^C. The pellet will then be re-suspended in 200μL 0.32M sucrose solution and stored under liquid nitrogen until analysis with the other blood fractions as one batch per volunteer.

**Erythrocyte and plasma separation**

Blood samples for erythrocyte and plasma selenoenzyme activity measurements will be transferred to EDTA coated blood tubes and centrifuged at 1210g at room temperature for 10 minutes. The plasma layer will be carefully removed and transferred to two 1.5mL cryovials for storage under liquid nitrogen. Isotonic saline (0.9%) solution will then be used to wash the remaining erythrocytes in the tube twice, using gentle tube inversion followed by centrifugation at 1210g at 4^o^C for 12 minutes. The erythrocytes will then be transferred to two 1.5mL cryovials and stored under liquid nitrogen.

**Determination of Protein Concentration**

Determination of the amount of protein in blood samples will be carried out using the dye binding assay of Bradford (1976) with a commercial source of human serum albumin (HSA) as the standard. The micro protein assay will be used; briefly 1mL of Bradford reagent will be added to 1mL of ~150 fold diluted sample and after 15 min the absorbance will be measured at 595 nm.

**Analysis of GPx1 activity**

GPx1 activity in blood samples (erythrocyte fraction) will be measured with hydrogen hydroperoxide as the substrate (Wendel 1981). The assay mixture will contain 25mM phosphate buffer pH 7.4, 2mM diaminoethanetetra-acetic acid (EDTA), 1mM sodium azide (NaN_3_), 3mM GSH, 0.2mM NADPH, 3U/ml yeast glutathione reductase, 0.1mM hydrogen peroxide and the appropriate amount of sample (e.g. 10μL). The reaction will be carried out at 37^o^C and oxidation of NADPH will be followed spectrophotometrically at 340nm.

**Analysis of glutathione peroxidase (GPx) activity**

GPx activity in blood samples will be measured essentially as described by Paglia and Valentine, 1967. All samples (erythrocytes, plasma and platelets) from one individual will be analysed in a single batch using a human reference serum sample (Seronorm) as an internal reference standard. Plasma samples will be added directly to the assay mixture without prior dilution whereas reconstituted erythrocytes (25μL) will be diluted and incubated for 5 mins with 500μL Drabkin’s reagent prior to addition to the assay. Platelet samples will be sonicated in ice-cold 50mM phosphate buffer pH 7.2 containing 4mM EDTA followed by centrifugation at 10000rpm, 4^o^C, the supernatant will then be diluted and treated with Drabkin’s reagent prior to assay. The reaction mixture will contain 1mM phosphate buffer pH 7.0, 0.01mM EDTA, 1U glutathione reductase, 5μM glutathione, 0.25μM NADPH, 2.5μM tert-butyl hydroperoxide and 100μL sample. The reaction will be carried out at 37^o^C and absorbance readings will be recorded at 340nm.

**Analysis of GPx4 activity**

GPx4 activity of blood samples will be determined using a sensitive and specific HPLC method as described by Bao et al., 1995. The substrate, 1-palmitoyl-2-(13-hydroperoxy-cis-9,trans-11-octadecadienoyl)-L-3-phosphatidylcholine (PLPC-OOH) will be prepared from PLPC using soybean lipoxidase as described by Maiorino et al., 1990. Briefly, PLPC-OOH will be separated from unoxidised phospholipid by FPLC on a PepRPC HR10/10 column with a gradient of water (100%) to methanol (100%). The concentration of the phospholipid hydroperoxide collected in 100% methanol will be determined by absorbance at 232 nm (ε = 25,000 M^-1^ cm^-1^) (Chan, 1984). 1-palmitoyl-2-(13-hydroxy-*cis*-9, *trans*-11-octadecadienoyl)-L-3-phos-phatidylcholine (PLPC-OH) standards will be prepared as described by Bao et al., 1995.

The GPx4 activity assay mixture will contain 0.1M Tris-HCl, pH 7.4, 2mM diaminoethanetetra-acetic acid (EDTA), 1mM sodium azide (NaN_3_), 3mM GSH, 0.12 % Triton X-100, 25μM PLPC-OOH and the appropriate amount of plasma (e.g. 10 μL) in a final volume of 0.5mL. The reaction will be carried out at 37^o^C for approximately 20 min and termination achieved by the addition of ice-cold acetonitrile. Prior to HPLC analysis the samples will be centrifuged at 11,600 g for 2 min. An Ultracarb 5 ODS (20) column (250 × 4.6 mm) or other suitable C18 column will used to separate PLPC-OOH and PLPC-OH with a mobile phase of acetonitrile, methanol and water (50:49.5:0.5, by vol.) containing 10mM choline chloride. The flow rate will be maintained at 0.5mL/min and detection at 232 nm. Conversion of substrate to product will be determined from the peak height of PLPC-OH standards.

**Analysis of thioredoxin reductase (TrxR) activity**

TrxR activity in plasma will be measured as described by Holmgren & Bjornstedt (1995) using insulin as the substrate. The TrxR assay mixture will contain 80μM HEPES pH 7.6, 3mM EDTA, 0.7μM NADPH, 0.4μM insulin, 30μM Trx and 70µL plasma (diluted 1:5 in water). The reaction will be incubated at 37^o^C for approx 20 min and then stopped with 1mM 5,5’-dithiobis-nitrobenzoic acid/6M guanidine hydrochloride in 0.2M Tris–HCl (pH 8.0), the absorbance will be recorded at 412nm. The absorbance of the control (all assay components except Trx) will be subtracted from the absorbance of the sample and TrxR activity will be calculated based on the standard curve prepared using pure TrxR.

**Determination of key Se-responsive protein levels**

Key Se-responsive protein levels will be analysed using appropriate proteomic techniques and using Western blotting techniques. Proteins from blood will be separated from blood using sodium-dodecyl sulphate polyacrylamide gel electrophoresis (SDS-PAGE) on a 12% NuPAGE Novex Bis-Tris gel (Invitrogen) and transferred to PVDF membrane (Millipore) following the manufacturer’s instructions. The membrane will be saturated for 1 hour at room temperature with Starting Block (TBS) blocking buffer (Pierce). Primary antibodies specific to Selenoprotein W1, Selenoprotein P and other key Se-responsive proteins will be used separately to probe the membrane, followed by a secondary horseradish peroxidase antirabbit IgG (Sigma). Protein bands will be visualised using chemoluminescence with a Supersignal West Pico Chemiluminescent substrate kit (Pierce) on a Fluor-S MultiImager (BioRad).

**RNA isolation from blood samples**

RNA will be extracted from whole blood and lymphocytes. For whole blood total RNA will be isolated using Tri Reagent BD (Molecular Research Centre) following manufacturer’s protocol. Lyphocytes will be isolated by diluting whole blood with 1 volume of phosphate buffered saline (PBS). Then a layer of 2 volumes of diluted blood will be placed on to 1 volume of Lymphoprep (Nycomed, UK) and centrifuged at 200g for 20 minutes at room temperature. The lymphocyte layer is removed and diluted >2 fold with PBS and centrifuged at room temperate for another 20 minutes at 200g. The supernatant is decanted off the remaining cell pellet. Total RNA is then extracted from the cells (after homogenization with a QIAshredder column) using Qiagen RNeasy mini kit according to the protocol described by the manufacturer (Qiagen, UK). RNA will be eluted from the column with RNase-free water and RNase inhibitor added immediately (20 U/preparation) and stored at -80°C. The concentration and purity of the RNA will be determined by measurement of the absorbance at 260 and 280nm and RNA 6000 Nano assay (Agilent Technologies) according to manufacturer’s instructions.

**Micro array analysis of global gene expression**

Affymetrix Gene Chip array expression profiling will carried out using the facilities at the John Innes Genome Lab, [http://www.jicgenomelab.co.uk](http://www.jicgenomelab.co.uk/). All protocols described can be found in the Affymetrix Expression Analysis Technical Manual II (Affymetrix Manual II) <http://www.affymetrix.com/support/technical/manuals.affx>. Briefly, first strand cDNA synthesis will performed according to the Affymetrix Manual II, using 5μg of total RNA. Second strand cDNA synthesis will be performed with minor modifications to the Affymetrix Manual II, double-stranded cDNA products will be immediately purified following the “Phase Lock Gel Cleanup of Double-Stranded cDNA” protocol and then the cDNA will be re-suspended in 22μL of RNAse free water.

cRNA preparation will be performed with minor modifications to the Affymetrix Manual II. Briefly, 11uL of cDNA will be used as a template to produce biotinylated cRNA using half the recommended volumes of the ENZO BioArray High Yield RNA Transcript Labelling Kit. Labelled cRNAs will be purified following the “Qiagen RNeasy Cleanup and Quantification of Biotin-Labelled cRNA” protocol (Affymetrix Manual II). cRNA quality will be assessed using Agilent RNA6000nano LabChips® (Agilent Technology 2100 Bioanalyzer Version A.01.20 SI211). cRNA quantity will be assessed by running 1μl of each cRNA sample on a NanoDrop® ND-1000 UV-Vis Spectrophotometer (Nanodrop Technologies). 20µg of cRNA will be fragmented and hybridisation cocktails will be setup according to the Affymetrix Manual II.

High-density oligonucleotide arrays containing probes of more than 22000 different human genes (human U133A arrays, Affymetrix, Santa Clara, CA) will be used for gene expression detection. Hybridization overnight at 45^o^C and 60RPM (Hybridization Oven 640), washing and staining (GeneChip® Fluidics Station 450, using the EukGEws2_450 Antibody amplification protocol) and scanning (GeneArray® 3000) will carried out according to the Affymetrix Manual II. CGOS (Affymetrix) will be used for image analysis and to determine probe signal levels. The average intensity of all probe sets will be used for normalization and scaled to 100 in the absolute analysis for each probe array. Data from GCOS will then be analysed in GeneSpring® software version 6.0 (Silicon Genetics, Redwood City, CA).

**Analysis of Key Se-responsive genes - RT-PCR**

**(TaqMan analysis)**

Determination of mRNA levels of key selenium responsive genes will be performed using real-time RT-PCR (TaqMan assay). Fluorogenic probes with 5’ FAM (6-carboxyfluoroscein) reporter dye and 3’ TAMRA (6-carboxytetramethylrhodamine) quencher dye will be used to target key selenium responsive genes, which may include (SepW1, SelP, Casp10, IL10, IGF1R, TNFS14, CD1D). RT-PCR reactions will be performed in 96 well plates using TaqMan one-step RT-PCR master mix reagent kit (Applied Biosystems) in a total volume of 25 µL/well consisting of 100nM probe, 200-300nM forward and reverse primers and 10ng RNA. TaqMan RT-PCR conditions: 48°C 30 min, 95°C 10 min then 40 cycles of 95°C for 15 s and 60°C for 1 min. TaqMan threshold cycle number (Ct) will be normalized into fold of relative induction using the equation of ∆Ct method, fold of induction = 2^Ct(control) – Ct(treatment)^. Standard curves will be constructed using total RNA in triplicate to provide a range which encompasses the range of observed sample values.

**6.62ii** Blood: **Cellular and Humoral Immune Response**

**NK- and CD8-mediated cell cytotoxicity**

NK and cytotoxic T cells (effector cells) will be isolated by negative selection. Target cell membranes will be labelled with a fluorescent carbocyanin dye. Effector and Target cells will be mixed together to allow cytotoxic reactions to occur. Propidium Iodide will then be added to this mixture to assess cell death. Quantification beads will be added at the same time. The first set of data will be acquired by flow cytometry to give an absolute viability count. The same cells will then be washed, fixed and permeabilised to allow detection of intracellular antigens after staining with fluorochrome-labelled phenotypic markers and antibodies to perforin and granzyme contained within the Effector cells. After washing the cells data will be acquired by flow cytometry.

**In vitro analysis of cytokines production**

Mononuclear cells will be isolated from peripheral blood by density gradient centrifugation and cultured under standard conditions in the presence or absence of influenza antigens. Supernatants will be collected and stored -20°C for later analyses of cytokine content. Secreted cytokines will be measured using bead array technology and according to manufacturer’s instructions. Data will be acquired by flow cytometry. A variant of this method will be used to detect levels of IFNγ. In such a case a protein transport inhibitor will be added 4 hours before termination of culture, after which cells will be washed, permeabilised and stained for intracellular cytokine and phenotype. Data will be acquired by flow cytometry.

**Tissue culture and bi-parametric DNA/BrdU analysis for flu-specific proliferative response**

Standard methods require the inclusion of a control antigen, i.e. polio-virus antigen. In order to develop this assay a prescription for obtaining the commercially available poliomyelitis vaccine (inactivated) will be sought from one of the IFR medical advisor. T-cells will be cultured in the presence or absence of influenza and polio antigens, according to standard methods. Bromodeoxyuridine (BrDU) will be added shortly before the end of the culture period. After a further incubation, BrDU-pulsed cells will be stained with fluorochrome-labelled antibodies for their phenotypic identification. These cells will then be washed, fixed, permeabilised and subject to DNAase treatment to expose incorporated BrDU before staining with fluorochrome-labelled anti-BrDU antibody. At this time cells may also be stained with other antibodies for detection of relevant intracellular proteins. Total DNA will be identified with a DNA-specific dye. All data will be acquired by flow cytometry.

**Isotype-specific antibody titres by ELISA**

96-well immunoplates will be coated with the antigen at 4C overnight, and then plates will be incubated with blocking solution (10%BSA in PBS) and then washed. Serum samples are then added in triplicate at appropriate dilutions. Samples of previously defined positive control sera and appropriate negative controls are run in each plate. Plates are incubated at room temperature for 1h and washed carefully. This is step is the followed by the incubation with unlabelled antibody specific to human isotype (IgA, IgG1 and IgG3) and then by incubation with peroxidase conjugated anti-mouse IgG. After washing, 200uL of the chromogenic substrate TMB are added to the wells. Reaction is stopped by addiction of acid, and the colour developed is read at 450nm using a microplate reader.

**Analysis of expression and distribution of NF-kB**

Expression and intracellular distribution of NF-κB and its inhibitor I-κBα will be evaluated by flow cytometry. After a recall challenge *in vitro* with influenza vaccine cells will be fixed and permeabilized using appropriate commercially available solution (Fix and Perm, Caltag, Medsystem Ltd) and then stained with specific anti-NF-κB and anti-IκBα antibodies. A mixture of fluorescently labelled (PE or Cy5) respectively will be then added. The samples will be then stained with propidium iodide and analysed by flow cytometry.

### 6.64 Saliva Samples: Humoral Response

**Detection of individual antibody forming cells by Enzyme linked immunospot (ELISPOT)**

Nitrocellulose filters will be coated with the appropriate dilution of viral protein or control antigen diluted in phosphate buffer saline. After incubation at 4^o^C overnight, plates are washed 3x with 300µL PBS-T per well. Background controls consisting in wells incubated under the same conditions but with PBS only are included in each plate. Plates are blocked with 200µL /well of complete medium for 30min-1h at 37^o^C and rinsed in PBS. This is followed by incubation with lymphocytes at different concentration for 4-6 hours at 37^o^C. Individual antibody forming cells (AFC) will be then detected by the addition of enzyme-labelled antibodies specific for different isotypes (IgA/G/M) Immunospots will be by the addiction of appropriated enzyme (alkaline phosphatase or HRP)-labelled antibody. Immunospot will then visualised by the addition of the appropriate enzyme colour development reagents. The substrate reaction will be stopped by rinsing and filters will be air-dried the spots enumerated either automatically or manually by inspection under dissecting microscope.

**Isotype-specific antibody titres by ELISA**

96-well immunoplates will be coated with the antigen at 4C overnight, and then plates will be incubated with blocking solution (10%BSA in PBS) and then washed. Saliva samples are then added in triplicate at appropriate dilutions. Samples of previously defined positive control sera and appropriate negative controls are run in each plate. Plates are incubated at room temperature for 1h and washed carefully. This is step is the followed by the incubation with unlabelled antibody specific to human isotype (IgA, IgG1 and IgG3) and then by incubation with peroxidase conjugated anti-mouse IgG. After washing, 200uL of the chromogenic substrate TMB are added to the wells. Reaction is stopped by addiction of acid, and the colour developed is read at 450nm using a microplate reader.

**6.65 Determination of alpha-tocopherol in serum**

Plasma carotenoids, retinol, and α-tocopherol will be assayed with a modified HPLC technique as descried by Hughes et al (1997). Duplicate aliquots (500μl) of freshly thawed plasma will be added to aqueous sodium dodecyl sulphate (lysing agent; 500μl; 10mmol/L), and retinol, α-tocopherol, and the carotenoids will be extracted simultaneously into two 3ml portions of hexane. The combined extracts will be dried under nitrogen and redissolved in dichloromethane and mobile phase mixture at a ratio of 1:9 (vol/vol) before HPLC analysis as described by Fuller et al (1992). Retinol, α-tocopherol, and the carotenoids will be determined simultaneously by monitoring the HPLC column eluent at 328nm (retinol) for the first 5 minutes of the run and at 297nm (α-tocopherol) for the following 10 minutes. Standard solutions for retinol and α-tocopherol will be combined with the mixed carotenoid working standard to give final concentrations of 2.5mg/L retinol and 100mg/L α-tocopherol.

# 7.0 Statistical Analysis

Power calculations were done so that, out of the six study groups, a difference of 1 standard deviation in response variable between the minimum and maximum responding groups would be reliably detected. For example, for plasma selenium, previous studies (Shortt et al, 1997) suggest that the population standard deviation is 11.7, and so our sample sizes are designed to give an 80% chance of detecting a range of plasma selenium between the six groups of 11.7. For antibody secreting cells/500,000 lymphocytes and specific serum antibody (both at day 7 post-vaccination) the corresponding standard deviations are 76 and 19 respectively (Cox et al, 1999).

The power calculations also assumed that any effect of selenium supplementation would be proportional to the amount of supplementation, with enriched onions being equivalent to a supplementation of 50µg/day selenium and the un-enriched onions equivalent to no supplementation.

The power calculations were also based on the assumption that the data would be analysed using a one-way ANOVA model, the grouping having six levels (control, 50µg/day selenium, 100µg/day selenium, 200µg/day selenium, enriched onions, non-enriched onions). In order to be able to detect a range of 1 standard deviation between the groups with a power of 80%, a sample size of 144 (or 24 in each group) is sufficient. This allows for a drop-out rate of 25%, or in other words, finishing with 18 subjects in each group.

In the unlikely event of there being a year effect, and thus the two onion groups (but not the control and supplementation groups) being potentially compromised, 24 subjects in each of the remaining 4 groups gives a power of 80% of detecting a range of 1 standard deviation in the response variable. This is based on using a two-way ANOVA model to analyse the data, with a three level year effect and a four level group effect (control, 50µg/d Se, 100µg/d Se, 200µg/d Se). It is important to stress however that we are aiming to recruit the full complement of 24 volunteers without the drop out rate of 25% as we feel there is a greater chance of compliance with the supplementation intervention.

In order to allow for the unlikely possibility of a year effect the control group and each of the three selenium supplementation groups will be evenly and randomly spread over the three years. Unfortunately due to time constraints, this is not possible with the onion groups which will be evenly and randomly spread over only two of the years (i.e. half in each year). The two onion groups will also be able to be analysed separately, but with a reduced power of 18 subjects per group gives a power of 80% of detecting a difference in response variable of 1 standard deviation. This is quite a large effect of the enriched onions, given that they are only “equivalent” to a selenium dose of 50µg/day. This is based on a 25% drop out rate expected for the onion intervention study.

The data would be initially analysed using an ANOVA model to test for differences in end-points between the groups. If the required data assumptions for this model are not met (e.g. normality), then data transformations and/or non-parametric methods will be employed. A repeated measures analysis using all 5 data points per subject will also be used, although it is the end-point (week 12) that is of primary interest.

**8.0 References:**

Bajer AA, Garcia-Tapia D, Jordan KR, Haas KM, Werling D, Howa CJ, Estes DM. Peripheral blood derived DC promote IgG restricted B cell responses in vitro. J Leukoc Biol 2003;73: 100-106.

Bao YP, Chambers SJ and Williamson G. Direct separation of hydroperoxy- and hydroxy-phosphatidylcholine derivatives: application to the assay of phospholipid hydroperoxide glutathione peroxidase. Anal Biochem 1995;224:395-399.

Bellingham J, Gregory-Evans K, Fox MF and Gregory-Evans CY. Gene structure and tissue expression of human selenoprotein W, SEPW1, and identification of a retroprocessed pseudogene, SEPW1P. Biochim Biophys Acta 2003;1627:140-146.

Belshaw N. J., Elliott G. O. & Johnson I. T. Use of DNA from human stools to detect aberrant CpG island methylation of genes implicated in colorectal cancer
Cancer Epidemiology Biomarkers and Prevention
2004, 13 1495-1501

Broome CS, McArdle F, Kyle JAM *et al.*  An increase in selenium intake improves immune function and poliovirus handling in adults with marginal selenium status. Am J Clin Nutr (in press).

Ca XJ, Block E, Uden PC, Xhang X, Quimby BD, Sullivan JA. Allium chemistry:

identification of selenoamino acids in ordinary and selenium-enriched garlic, onion and broccoli using gas chromatography with atomic emission detection. J Agric Food Chem 1995;43:1754-7.

Cakman I, Rohwer J, Schutz *et al.* Dysregulation between TH1 and TH2 T cell subpopulations in the elderly. Mech Ageing Dev 1996;87:197-209.

Center for Diseases Control and Prevention. Public health and aging: influenza vaccination coverage among adults aged >or = 50 years and pneumococcal vaccination coverage among adults aged > or = 65 years- United States, 2002. MMWR Morb Mortal Wkly Rep 2003;52:987-92

Clark LC, Combs GRJr, Turnball BW *et al.* Effects of selenium supplementation for cancer prevention in patients with carcinoma of the skin. JAMA 1996;276:1957-63.

Combs G F, Selenium in global food systems. Brit J Nut 2001; 85, 517-547

Cox RJ, Mykkeltveldt E, Sjursen H, Haaheim LR. The effect of zanamir on the early immune response to influenza vaccination. Vaccine1999;19:4743-49.

Cox RJ, Mykkeltveldt E, Sjursen H, Haaheim LR. The effect of zanamir on the early immune response to influenza vaccination. Vaccine1999;19:4743-49.

Delves, H.T., and Sieniawska, C.E. Simple Method for the Accurate Determination of Selenium in Serum by Using Inductively Coupled Plasma Mass Spectrometry. *JAAS*. 1997**, 12**, 387 – 389

Finch, JM., Turner, RJ. Effects of Selenium and vitamin E on the immune responses of domestic animals. Res Vet Sci, 1996; 60:97-106.

Fox, T. The use of stable isotopes for studying selenium metabolism in humans. PhD thesis, University of East Anglia, 2002;pp57-60.

Fuller CJ, Faulkner H, Bendich A *et al* Effect of beta-carotene supplementation on photosuppression of delayed-type hypersensitivity in normal young men. Am J Clin Nutr 1992; 56: 684-90.

Gammelgaard, B., Bendahl, L., Sidenius, S. and Jøns, O. Selenium speciation in urine by ion-pairing chromatography with perfluorinated carboxylic acids and ICP-MS detection. *JAAS*. 2002**, 17**, 570 – 575.

Gardner EM, Bernstein ED, Popoff KA *et al.* Immune response to influenza vaccine in healthy elderly: lack of association with plasma β-carotene, retinol, α-tocopherol, or zinc. Mechanisms of Ageing and Development 2000;117:29-45.

Ge H, Cai X-J, Tyson JF, Uden PC, Denoyer ER and Block E. Identification of selenium species in selenium-enriched garlic, onion and broccoli using high-performance ion chromatography with inductively coupled plasma mass spectrometry detection. Anal Comm 1996;33:279-281.

Girodon F, Galan P, Monget AL *et al.* Impact of trace elements and vitamin supplementation on immunity and infections in institutionalized elderly patients:a randomized controlled trial. Arch Intern Med 1999: 159, 748-54.

Govaert TME, Sprenger MJW, Dinant GJ *et al*. Immune response to influenza vaccination in elderly people. A randomized double-blind placebo controlled trail. Vaccine 1994;12: 1185-89.

Gu Q-P, Beilstein MA, Barofsky E, Ream W and Whanger PD. Purification, characterisation and glutathione binding to selenoprotein W from monkey muscle. Arch Biochem Biophys1999; 361:25-33.

Gu Q-P, Sun Y, Ream LW and Whanger PD. Selenoprotein W accumulates primarily in primate skeletal muscle, heart, brain and tongue. Mol Cell Biochem 2000; 204:49-56.

Holmgren A and Bjornstedt M. Thioredoxin and thioredoxin reductase. Methods Enzymol. 1995;252:199-208.

Hughes DA, Wright AJA, Finglas PM *et al* The effect of β-carotene supplementation on the immune function of blood monocytes from healthy male nonsmokers. J Lab Clin Med 1997; 129: 309-317.

Ip C, Ganther H. Activity of methylated forms of selenium in cancer prevention. Cancer Res 1996;50:1206-11.

Ivory K, Martin R, Hughes DA. Significant presence of terminally differentiated T cells and altered NF-kB and I-kBa interactions in healthy ageing. Exp Gerontology (in press).

Ivory K, Martin R, Tomkies K, Waldron K, Highes DA. Natural killer cell cytotoxicity: simultaneous quantitative analysis of conjugate formation, degranulation and killing by multi-colour flow cytometry. Immunology 2001;104 (S1):119-20.

Jeong D, Kim TS, Chung YW, Lee BJ and Kim IY. Selenoprotein W is a glutathione- dependent antioxidant in vivo. FEBS Lett. 2002;517:225-228.

Jens J. Sloth, J.J., Larsen, E.H., Bügelb, S.H., and Moesgaard, S. Determination of total selenium and 77Se in isotopically enriched human samples by ICP-dynamic reaction cell-MS. *JAAS* 2004**, 18**, 317 – 322.

Keitel WA, Cate TR, Couch RB. Efficacy of sequential annual vaccination with inactivated influenza virus vaccine. 1988. Am J. Epidemiol 1988;127:353-64.

Labat L, Dehon B and Lhermitte M. Rapid and simple determination of selenium in blood serum by inductively coupled plasma-mass spectrometry (ICP-MS) Anal and Bioanal Chem. 2003;376:270-273.

McElhalney JE, Meneilly GS, Lechelt KE *et al*. Antibody response to whole virus and split virus influenza vaccines in successful ageing. Vaccine 1993;10:1055-60.

McKenzie RC., Rafferty TS., Beckett GJ. Selenium: an essential element for immune function. Immunol Today, 1998; 19:342-5.

Marchante-Gayon JM, Feldmann I, Thomas C and Jakubowski N. Speciation of selenium in human urine by HPLC-ICP-MS with a collision and reaction cell. J Anal At Spectrom

2001;16:457-463.

Muszkat M, Greenbaum E, Ben-Yehuda A *et al*. Local and systemic immune response in nursing-home elderly following in or im immunization with inactivated influenza vaccine. Vaccine. 2003;21:1180-86.

Nicholson KG, Wood JM, Zambon M. Influenza. Lancet 2003; 362:1733-45.

Nicoletti C, Borghesi C. Detection of autologous anti-idiotypic AFC by a modified ELISPOT. Res Immunol 1992;143:919-25.

Nicholson KG, Wood JM, Zambon M. Influenza. Lancet 2003;362:1733-45.

Paglia DE and Valentine WN. Studies on the quantitative and qualitative characterisation of erythrocyte glutathione peroxidase. J Lab Clin Med 1967;70:158-169.

Philipp S, Strauss B, Hirnet D et al. TRPC3 mediates T-cell receptor-dependent calcium entry in human T-lymphocytes. J Biol Chem 2003;278:26629-26638.

Ravaglia G, Forti P, Maioli F *et al.* Effect of micronutrient status on natural killer cell function in healthy free-living subjects. Am J Clin Nutr 2000;71:590-8.

Rayman MP. Dietary selenium: time to act. British Medical Journal 1997;314:387-8.

Rayman MP. The Importance of selenium to human health. Lancet 2000;356:233-4.

Reilly C. Selenium in Food and Health. London: Chapman & Hall, 1996.

Shah, M., Kannamkumarath, S.S., Wuilloud, J.C.A., Wuilloud, R.G., and Caruso, J.A. Identification and characterization of selenium species in enriched green onion (Allium fistulosum) by HPLC-ICP-MS and ESI-ITMS. *JAAS*. 2004**, 19**, 381 – 386.

Shortt CT, Duthie GG, Robertson JD, Morrice PC, Nicol F, Arthur J. Selenium status of a group of Scottish adults. EJCN 1997;51:400-404.

Solana R, Alonso MC, Pena J. Natural killer cells in healthy aging. Exp. Gerontology. 1999: 34, 435-43. Standing Committee on the Evaluation of Dietary Reference Intakes of the Food and Nutrition Board, Institute of Medicine, National Academies and Health Canada. Dietary Reference Intakes for Vitamin C, Vitamin E, Selenium and Carotenoids, Washington, DC: National Academy Press, 2000.

Taylor EM., Selenium and cellular immunity. Evidence that selenoproteins may be encoded in the +1 reading frame overlapping the human CD4, CD8 and HLA-DR genes. Biol Trace Elem Res 1995; 49:85-95.

Thompson WW, Shay DK, Weintraub E, Brammer L, Cox N, Ander LJ, Fukuda K. Mortality associated with influenza and respiratory suncitial virus in the US. JAMA 2003;289:179- 86.

Tujebajeva RM, Harney JW, and Berry MJ. **Selenoprotein P Expression, Purification, and Immunochemical Characterization.** J Biol Chem 2000;275:6288-6294.

Whanger PD. Selenoprotein W: a review. Cell Mol Life Sci 2000; 57: 1846-1852.

Whanger PD. Selenium and its relationship to cancer: an update. Br J Nutr 2004;91:11-28.

Wood SM, Beckham C, Yosioka A, Darban H, Watson RR. Beta carotene and selenium supplementation enhances immune responses in aged humans. Integr Med 2000;2: 85-92.

Yoshizawa K, Willett WC, Morris SJ *et al.* Study of prediagnostic selenium level in toenails and the risk of advanced prostate cancer. J Natl Cancer Inst 1998;90:1219-24.

Ysart G, Miller P, Crews H *et al.* Dietary exposure estimates of 30 elements from the UK Total Diet Study. Food Addit Contam 1999;16:391-403.

Zeng H, Davis CD, Finley JX. Effect of selenium-enriched broccoli diet on differential gene expression in min mouse liver. J Nutr Biochem 2003;14:227-231.

Zhang JS, Svehlikova V, Bao YP, Howie AF, Beckett, GJ and Williamson G. **Synergy between sulforaphane and selenium in the induction of thioredoxin reductase 1 requires both transcriptional and translational modulation**. Carcinogenesis 2003;24:497-503.
