# Supplementary material for: Effects of Selenium Supplementation on Selenoprotein Gene Expression and Response to Influenza Vaccine Challenge: A Randomised Controlled Trial
Source: PLoS One. 2011 Mar 21;6(3):e14771. doi: 10.1371/journal.pone.0014771 (PMC3061857; doi:10.1371/journal.pone.0014771)
Supplement: Text S1 — Approval Letter from Norwich Ethics Committee (0.08 MB DOC) [file pone.0014771.s002.doc]

**Text S1.** Approval from Norwich Ethics Committee.

Norwich Research Ethics Committee

c/o The Norfolk & Norwich University Hospital NHS Trust

First Floor

Aldwych House

57 Bethel Street

Norwich

NR2 1NR

19 April 2005

Professor Susan Fairweather-Tait

Head of Nutrition Division

Institute Of Food Research,

Norwich Research Park,

Colney

Norwich

NR4 7UA

Dear Professor FAirweather-Tait

| ***Full title of study:*** | ***Selenium Status and Immune Function*** |
| --- | --- |
| ***REC reference number:*** | ***05/Q0101/32*** |
| ***Protocol number:*** | ***Version 3, March 2005*** |
|  |  |

Thank you for your letter of 23 March 2005 from Dr Charlotte Armah, responding to the Committee’s request for further information on the above research and submitting revised documentation.

The further information has been considered on behalf of the Committee by the Chair in consultation with Mrs Belinda Hoste and Miss Rosemary Jackson.

## **Confirmation of ethical opinion**

On behalf of the Committee, I am pleased to confirm a favourable ethical opinion for the above research on the basis described in the application form, protocol and supporting documentation as revised.

# Conditions of approval

The favourable opinion is given provided that you comply with the conditions set out in the attached document. You are advised to study the conditions carefully.

## **Approved documents**

The final list of documents reviewed and approved by the Committee is as follows:

| **Document Type:** | **Version:** | **Dated:** | **Date Received:** |
| --- | --- | --- | --- |
| Application | Parts A, B | 22/02/2005 | 23/02/2005 |
| Application | Part C | 01/02/2005 | 23/02/2005 |
| Investigator CV | Susan J Fairweather-Tait |  | 23/02/2005 |
| Protocol | 3: Annex 1 | 01/03/2005 | 29/03/2005 |
| Covering Letter |  | 23/02/2005 | 23/02/2005 |
| Advertisements | 3: Annex 3 | 01/03/2005 | 23/02/2005 |
| Letters of Invitation to Participants | 2: Annex 2 | 01/02/2005 | 23/02/2005 |
| GP/Consultant Information Sheets | 2: Annex 9 | 01/02/2005 | 23/02/2005 |
| Participant Information Sheet | 3: Annex 4 | 01/03/2005 | 29/03/2005 |
| Participant Consent Form | 3: Annex 5a | 01/03/2005 | 29/03/2005 |
| Response to Request for Further Information |  | 23/03/2005 | 29/03/2005 |
| Revised response to A17, C3 |  |  | 29/03/2005 |
| Medication / medical conditions declaration agreement | 2: Annex 6 | 01/02/2005 | 23/02/2005 |
| Screening Questionnaire | 2: Annex 7 | 01/02/2005 | 23/02/2005 |
| Urine dip stick test results form | 2: Annex 8 | 01/02/2005 | 23/02/2005 |
| Clinical test results form | 2: Annex 8 |  | 23/02/2005 |
| Letter to GP – Re: blood results | 2: Annex 10 | 01/02/2005 | 23/02/2005 |
| Letter to GP – abnormal results | Annexe 11 | 01/02/2005 | 23/02/2005 |
| Letter to volunteer – abnormal results | 2: Annex 12 |  | 23/02/2005 |
| Letter to volunteer – advised not to have flu vaccination | 2: Annex 13 | 01/02/2005 | 23/02/2005 |
| Registration Form for Human Studies (IFR) |  |  | 23/02/2005 |
| Letter from Linda Harvey to Prof Fairweather-Tait |  | 11/02/2005 | 23/02/2005 |
| Letter to Chair IFR Human Research Committee |  | 21/02/2005 | 23/02/2005 |
| IFR Human Research Committee approval letter |  | 22/02/2005 | 23/02/2005 |

## **Management approval**

The study should not commence at any NHS site until the local Principal Investigator has obtained final management approval from the R&D Department for the relevant NHS care organisation.

## **Notification of other bodies**

The Committee Administrator will notify the research sponsor that the study has a favourable ethical opinion.

### Statement of compliance

*The Committee is constituted in accordance with the Governance Arrangements for Research Ethics Committees (July 2001) and complies fully with the Standard Operating Procedures for Research Ethics Committees in the UK.*

# 05/Q0101/32 Please quote this number on all correspondence

With the Committee’s best wishes for the success of this project,

Yours sincerely,

**Janette R Guymer (Mrs)**

#####	REC Manager

E-mail: janette.guymer@nnuh.nhs.uk

#### Enclosures Standard approval conditions

***Site approval form (SF1)***
